# *N*-acetylglucosamine regulates the siderophore lysochelin production through inducing the *p*-hydroxybenzaldehyde biosynthesis in *Lysobacter* sp. 3655

**DOI:** 10.3389/fmicb.2026.1808803

**Published:** 2026-04-24

**Authors:** Lin Jiang, Xiaofang Yan, Fang Zhang, Ailing Chen, Yixuan Chen, Yongbiao Zheng, Lingjun Yu

**Affiliations:** School of Life Sciences, Fujian Normal University, Fuzhou, Fujian, China

**Keywords:** *Lysobacter*, lysochelin, *N*-acetylglucosamine, *p*-hydroxybenzaldehyde, regulation

## Abstract

**Introduction:**

*N*-acetylglucosamine (GlcNAc) serves as a signaling molecule that triggers a variety of physiological responses in microorganisms. However, its regulatory roles in metabolite biosynthesis and siderophore production in *Lysobacter* sp. 3655 remain unclear, especially regarding the quorum sensing (QS) signal 4-hydroxybenzoic acid (4-HBA) and the siderophore lysochelin under iron-deficient conditions. This study aimed to explore the regulatory effects of GlcNAc on metabolite profiles and lysochelin biosynthesis in *Lysobacter* sp. 3655.

**Methods:**

*Lysobacter* sp. 3655 was cultured in oligotrophic medium with GlcNAc induction. The induced differential metabolite was isolated and structurally identified by nuclear magnetic resonance and liquid chromatography-mass spectrometry. Gene deletion mutants of GlcNAc catabolic genes (*nagA*, *nagE2*), and L-phenylalanine biosynthesis gene (pheA) were constructed, as well as multiple deletion mutants in the *lenB2* (4-HBA biosynthetic gene) mutant background. Growth status and lysochelin yield of these mutants were evaluated under iron-deficient conditions. Exogenous complementation assays were performed using 4-HBA, GlcNAc, *p*-hydroxybenzaldehyde, cinnamic acid, and p-hydroxycinnamic acid to verify their functional effects.

**Results:**

GlcNAc specifically induced the production of a unique metabolite in *Lysobacter* sp. 3655, which was identified as *p*-hydroxybenzaldehyde. Deletion of *nagA*, *nagE2*, or pheA completely abolished GlcNAc-induced *p*-hydroxybenzaldehyde biosynthesis. The *lenB2* mutant showed significant growth defects and remarkably decreased lysochelin production under iron limitation, while exogenous 4-HBA or GlcNAc restored both phenotypes. Further disruption of *nagA*, *nagE2*, or *pheA* in the *lenB2* mutant eliminated the complementation effect of GlcNAc on lysochelin production. *p*-hydroxybenzaldehyde restored lysochelin biosynthesis by converting to 4-HBA, and the L-phenylalanine catabolic intermediates cinnamic acid and p-hydroxycinnamic acid also rescued the growth and lysochelin production defects of the *lenB2* mutant.

**Discussion:**

Our results reveal that GlcNAc regulates two metabolic pathways in *Lysobacter* sp. 3655: it promotes *p*-hydroxybenzaldehyde production through GlcNAc catabolism and L-phenylalanine metabolism, and restores lysochelin biosynthesis in the QS-deficient *lenB2* mutant via either 4-HBA converted from *p*-hydroxybenzaldehyde or alternative pathways mediated by L-phenylalanine catabolites. These findings uncover a novel regulatory network controlling lysochelin biosynthesis in *Lysobacter* sp. 3655, providing important references for investigating the biocontrol mechanisms of *Lysobacter* species.

## Introduction

Iron is an essential micronutrient for microbial growth, playing critical roles in key physiological processes including cellular respiration, DNA synthesis, DNA repair, and cellular antioxidant defense (Hantke, 2001). Under iron-deficient conditions, microorganisms biosynthesize and secrete siderophores—small, high-affinity iron-chelating molecules—to take up trace environmental iron and maintain normal growth (Hider and Kong, 2011). The high iron-chelating affinity of siderophores confers broad applicability in agricultural systems. Siderophores secreted by plant growth-promoting rhizobacteria (PGPR) have emerged as pivotal bioactive molecules for sustainable crop protection (Ansabayeva et al., 2025). By chelating Fe^3+^ into soluble ferric–siderophore complexes, these metabolites facilitate iron acquisition via plant transporters, such as Yellow Stripe-Like (YSL) proteins (Kobayashi and Nishizawa, 2012). Moreover, siderophores can solubilize other insoluble minerals (e.g., zinc, copper and manganese) into plant-available forms, thereby fostering plant growth (Mishra et al., 2023). It was found that a siderophore isolated from *Chryseobacterium* sp. C138 significantly promotes chlorophyll biosynthesis through increasing iron availability, and concomitantly enhancing tomato fruit yield (Radzki et al., 2013). Beyond nutrient mobilization, siderophores mediate biocontrol via nutritional immunity—a key antagonistic mechanism against phytopathogens (Wang et al., 2021). High-affinity siderophores, such as pyoverdines and bacillibactin, sequester available iron, generating iron-depleted microenvironments that suppress phytopathogen proliferation (Loper and Henkels, 1999). They often synergize with antimicrobial metabolites to improve biocontrol efficiency, as exemplified by *Pseudomonas putida* strains that suppress the growth of *Ralstonia solanacearum* and *Erwinia carotovora* (Chaudhari et al., 2017; Raaijmakers et al., 2010), which underscores their potential to reduce reliance on chemical fungicides in disease-suppressive soils (Danish et al., 2024).

Furthermore, siderophores enable their producers to gain a competitive growth advantage. When *Streptomyces venezuelae* and *Saccharomyces cerevisiae* are co-cultured, *S. venezuelae* produces the canonical siderophore desferrioxamine and the non-canonical siderophore foroxymithine, which are secreted at distinct subcellular locations (Shepherdson and Elliot, 2022). By chelating iron ions in the culture medium, the non-canonical siderophore foroxymithine establishes an iron-limited microenvironment surrounding *S. venezuelae*, thereby constraining the growth of *S. cerevisiae* (Shepherdson and Elliot, 2022). Additionally, the siderophore coelichelin, produced by *Streptomyces* sp. I8–5, inhibits the activation of the global transcription factor Spo0A in *Bacillus subtilis* (Zang et al., 2025). This inhibition disrupts the physiological homeostasis of *B. subtilis*, rendering it more susceptible to infection by bacteriophage SPO1 (Zang et al., 2025). Siderophores further support durable plant–microbe interactions by regulating biofilm formation and rhizosphere colonization (Jha et al., 2018). By modulating iron availability, they control biofilm-related gene expression, with siderophore-deficient mutants showing impaired biofilm development (Banin et al., 2005). Acting as signaling molecules, siderophores influence microbial motility and quorum sensing—traits critical for competitive root colonization, displacement of harmful microbes, and establishment of protective biofilms (Popat et al., 2017; Beneduzi et al., 2012).

Siderophore-producing PGPR, such as *Bacillus* spp. and *Pseudomonas* spp., have been demonstrated to be effective biological control agents (Rebouh et al., 2023). However, over the past two decades, *Lysobacter* has also emerged as a widely applied agent in biological control. For instance, Heat-stable antifungal factor (HSAF) produced by *Lysobacter enzymogenes*, a potential biocontrol agent, controlling Pear Valsa canker and wheat Fusarium head blight in fields (Hou et al., 2023; Zhao et al., 2019); ACC deaminase from *Lysobacter gummosus* OH17 promotes root development in *Oryza sativa* cv. Nipponbare (Laborda et al., 2018). Moreover, *Lysobacter fragariae* enhances Tomato fruit quality by the increment of fruit nutrition and functional components (Fu et al., 2025), while *Lysobacter firmicutimachus* exhibits strong biocontrol efficacy against Rice seedling disease caused by Pythium *arrhenomanes* in nursery trays (Tu et al., 2023). Nevertheless, few studies have focused on siderophores in *Lysobacter*. Lysochelin represents the first siderophore identified in this genus; however, the regulatory mechanisms underlying its biosynthesis remain uncharacterized (Miller et al., 2023). In our previous work, we demonstrated that *Lysobacter* sp. 3655 is also capable of producing lysochelin, and maltose induces lysochelin biosynthesis by promoting the high-density growth of the strain (Zhang et al., 2024).

The biosynthesis of microbial siderophores is tightly regulated by multiple mechanisms, with intracellular ferrous ion (Fe^2+^) as the primary control factor (Raymond et al., 2003). The Fur (ferric uptake regulator) system responds to Fe^2+^ concentrations and regulates siderophore biosynthesis in most bacterial species (Beauchene et al., 2015; Visca and Imperi, 2018). Furthermore, Fur integrates non-iron environmental cues: in *Acinetobacter baumannii*, photoreceptor BlsA interacts with Fur to activate siderophore acinetobactin biosynthesis in darkness/23°C, while blue light/30°C abrogates this interaction, restoring Fur-mediated repression (Tuttobene et al., 2018). Quorum sensing (QS), which mediates microbial intercellular communication via signal molecules to regulate collective behaviors (Waters and Bassler, 2005), broadly governs siderophore biosynthesis in various microorganisms. For instance, deleting *Pseudomonas aeruginosa* QS gene *lasR* or activating its QS-associated BfmRS two-component system enhances pyoverdine production (Stintzi et al., 1998; Song Y. et al., 2024), while the QS antagonist furanone stimulates pyoverdine biosynthesis in *P. aeruginosa* but inhibits the production in *P. putida* (Ren et al., 2005). In *Chromobacterium violaceum*, QS regulator CviR represses siderophores at high cell density (Batista et al., 2024), whereas *Vibrio harveyi* uses QS to differentially regulate siderophore types by cell density (McRose et al., 2018). Uropathogenic *Escherichia coli* (UPEC) produces yersiniabactin, a siderophore that acts as a QS molecule and accumulates via autoinduction at high cell density (Heffernan et al., 2024). QS-mediated siderophore regulation shows marked interspecific variability linked to survival strategies: some microbes upregulate biosynthesis at high cell density for conspecific use, while others repress it to redirect resources to growth (Darch et al., 2012).

*N*-acetylglucosamine (GlcNAc) is a key component of bacterial/fungal cell walls (peptidoglycan/chitin) and a signaling molecule regulating microbial physiology (Konopka, 2012; Naseem et al., 2012; Park and Uehara, 2008). It modulates secondary metabolism across various microbes: in *Streptomyces coelicolor*, GlcNAc induces antibiotics (undecylprodigiosin/actinorhodin) but represses siderophores (coelichelin/desferrioxamine) via DasR regulator (Rigali et al., 2008; Craig et al., 2012); it activates diverse metabolites including 3-formylindole, guaymasol, surfactin, bacillibactin in actinomycetes (Dashti et al., 2017), and bioactive compounds in sponge-associated *Streptomyces* (Alkhalifah, 2021), yet impairs erythromycin production in *Saccharopolyspora erythraea* (Liao et al., 2015). GlcNAc also enhances pyocyanin production in *P. aeruginosa* and bleomycin yield in *Streptomyces verticillus* (Korgaonkar and Whiteley, 2011; Chen et al., 2020). Moreover, it interacts with QS: inhibiting *C. violaceum* QS via LuxR inactivation and *luxR* repression (Kimyon et al., 2016), and forming a feedback loop with QS in *Yersinia pseudotuberculosis* via NagC regulator (Wiechmann et al., 2023). However, the functional links between GlcNAc, QS, and siderophore biosynthesis remain unclear.

In the study, we describe the effects of GlcNAc and QS signal (4-HBA) on the biosynthesis of the siderophore lysochelin in *Lysobacter* sp. 3655. We found that GlcNAc induces the production of *p*-hydroxybenzaldehyde in the strain when cultured in oligotrophic medium, which is associated with GlcNAc utilization and L-phenylalanine metabolism. Furthermore, deletion of the 4-HBA biosynthetic gene (*lenB2*) abrogated lysochelin production in the mutant when cultured in iron-deficient oligotrophic medium; conversely, exogenous supplementation with 4-HBA, GlcNAc, or *p*-hydroxybenzaldehyde restored lysochelin production in the *lenB2* mutant. Additional experiments revealed that exogenous *p*-hydroxybenzaldehyde is converted to 4-HBA in the *lenB2* mutant, suggesting that GlcNAc might regulate lysochelin biosynthesis through 4-HBA-mediated QS pathway. Collectively, this study provides novel insights into the regulatory network governing the siderophore lysochelin in *Lysobacter* sp. 3655, which respond to environmental carbon sources and QS signals.

## Materials and methods

### Bacterial strains, plasmids, and growth conditions

Bacterial strains and plasmids used in this study are shown in Supplementary Table S1. Luria-Bertani (LB) broth was used for the growth of *Lysobacter* sp. 3655 (wild-type, WT) and its derivative strains. M813 modified medium (M813m, 22.2 mM glucose, 17.2 mM K_2_HPO_4_, 10.0 mM NaH_2_PO_4_⋅H_2_O, 18.7 mM NH_4_Cl, 1.2 mM MgSO_4_, 2.0 mM KCl, 100 μM CaCl_2_, 10 μM FeSO_4_⋅7H_2_O), and M813m with 1% (w/v) GlcNAc or 10 mM L-phenylalanine was used for the *p*-hydroxybenzaldehyde production of the WT strain and its isogenic mutants. M813m-Fe medium (M813m without FeSO_4_⋅7H_2_O) was used for the growth, lysochelin production, and RNA extraction of the WT strain and its mutants. M813m-Fe medium supplemented with 3-HBA (50 μM), 4-HBA (50 μM), *p*-hydroxybenzaldehyde (50 μM), cinnamic acid (500 μM), *p*-hydroxycinnamic acid (500 μM) or L-phenylalanine (10 mM) were used to assess the yield of lysochelin and growth of the *lenB2* deletion mutant (Δ*lenB2*). M813m-Fe medium supplemented with purified lysochelin (50 μM) was used to assess the growth of the Δ*lenB2* strain. M813m-Fe medium supplemented with various concentrations of 4-HBA (final concentrations of 0, 20, 50, 100, and 200 nM) or GlcNAc (final concentrations of 0, 0.25, 0.5, 1, and 2%, w/v) was used to evaluate the effect of 4-HBA or GlcNAc on lysochelin production in the Δ*lenB2* strain. YME medium (4 g/L glucose, 10 g/L malt extract, 4 g/L yeast extract), 1/10 TSB medium (3 g/L tryptic soy broth), YME with 1% (w/v) GlcNAc, and 1/10 TSB with 1% (w/v) GlcNAc were used for detecting the production of *p*-hydroxybenzaldehyde in the WT strain. *E. coli* strain DH5α was cultured at 37°C in LB medium supplemented with gentamicin (Gm, 50 μg/mL) to propagate plasmids. *E. coli* strain S17-1 was used as the donor strain for intergeneric conjugation. Malt extract was purchased from Beijing Solabio Technology Co., Ltd. (Beijing, China), LB broth and other chemical reagents were purchased from Shanghai Aladdin Biochemical Technology Co., Ltd. (Shanghai, China).

### Primers and PCR

All primers used in this study are listed in Supplementary Table S2. PCR amplifications were conducted using either Phanta^®^ Max Super-Fidelity DNA polymerase (Vazyme) or rTaq DNA polymerase (Takara). For reactions with Phanta DNA polymerase, amplification started with an initial denaturation at 95°C for 3 min, followed by 30 cycles: denaturation at 95°C for 15 s, annealing at 60°C for 15 s, and elongation at 72°C for 1 min. The reaction was completed with a final extension at 72°C for 5 min. For rTaq DNA polymerase-mediated reactions, initial denaturation was performed at 95°C for 5 min, followed by 30 cycles of amplification: denaturation at 95°C for 30 s, annealing at 60°C for 30 s, elongation at 72°C for 1 min, and additional 10 min at 72°C.

### DNA manipulation and mutant construction

Chromosomal DNA and plasmids were isolated from *E. coli* DH5α, WT and its mutants using alkaline lysis method (Sambrook et al., 1989). NCBI protein database searches and sequence analyses were performed with the online tool PSI-BLAST (Altschul et al., 1997). To construct gene deletion mutants of *nagA* (*orf143*), *Nage2* (*orf5619*), *dasR* (*orf145*), and *pheA* (*orf2468*), gene-specific primer pairs were designed to amplify the upstream and downstream flanking regions of each target gene. For *nagA*, *Nage2*, *dasR*, and *pheA*, the upstream flanking fragments were 1,362, 1,200, 1,051, and 1,401 bp in length, while the downstream flanking fragments were 1,241, 1,031, 913, and 1,358 bp, respectively. Each set of upstream and downstream flanking fragments was double-digested with restriction enzymes and subsequently ligated into the corresponding sites of the plasmid pJQ200SK to construct recombinant plasmids. These plasmids were first introduced into *E. coli* S17-1 by heat shock transformation and subsequently transferred to WT or the Δ*lenB2* strain via intergeneric conjugation. In detail, WT (or Δ*lenB2*) and *E. coli* S17-1 were cultured in LB broth to the OD_600_ value of 0.30, respectively. Then 1 mL of each bacterial culture was collected and centrifuged to harvest the cell pellets. The pellets were resuspended separately in 100 μL MgSO_4_ (10 mM), mixed and streaked onto LB agar plates supplemented with 100 μg/mL kanamycin (Km) and 150 μg/mL gentamicin (Gm). After incubation at 30°C for 72 h, single-crossover colonies (plasmid integration into the chromosome via homologous recombination) were picked and cultured on LB agar plates containing 10% (w/v) sucrose (used for counterselection) and 100 μg/mL Km, followed by a second incubation at 30°C for 72 h. These colonies were then streaked onto two sets of LB agar plates: one with 100 μg/mL Km alone, and the other with a combination of 100 μg/mL Km and 150 μg/mL Gm. Colonies exhibiting resistance to Km but sensitivity to Gm were identified as potential double-crossover mutants and selected for PCR verification using the respective gene-specific primers.

### HPLC analysis and quantification of the differential compound and lysochelin

WT and its mutant derivatives were pre-cultured in 3 mL LB medium for 24 h, before being inoculated into 25 mL of either M813m medium, YME medium and 1/10 TSB medium supplemented with GlcNAc (for differential compound analysis) or M813m-Fe medium (for lysochelin analysis). All cultures were shaking at 30°C for 72 h, and each culture was mixed with an equal volume of butanol (supplemented with 1% trifluoroacetic acid) for extraction. The butanol fraction was concentrated to dryness under reduced pressure using a Buchi Rotavapor R-200, and the resulting crude extract was reconstituted in 1 mL of methanol. An aliquot (20 μL) of each reconstituted extract was subjected to high-performance liquid chromatography (HPLC) analysis using a Thermo UltiMate 3000 system equipped with a reversed-phase C18 column (Acclaim™ 120, 250 mm × 4.6 mm) and a UV detector set at 318 nm, with a constant flow rate of 1 mL/min. The mobile phase consisted of solvent A (water containing 0.05% formic acid) and solvent B (acetonitrile containing 0.05% formic acid), with the following gradient elution program: 5–25% B (0–5 min), increased to 80% B by 20 min (held for 1 min), further raised to 100% B (26–28 min), and returned to 5% B in the final 2 min. Quantification of the differential compound or lysochelin was calculated by normalizing the HPLC peak area to the OD_600_ value of the corresponding culture. Unless otherwise specified, the yield of the differential compound or lysochelin was expressed as the fold change relative to the yield of the respective control strain (e.g., the wild-type strain cultured in M813m or M813m-Fe medium). The experiments were repeated three times with three technical replicates each.

### Growth assay of WT and its mutants

WT was pre-cultured in 3 mL LB medium for 24 h, before being transferred to 250 mL Erlenmeyer flasks containing 25 mL M813m-Fe medium (liquid-air ratio was 1:9) and incubated with shaking (200 rpm) at 30°C for growth. Then 500 μL culture was transferred into quartz cuvette and a spectrophotometer (UV-8000, METASH) was used to determine OD_600_ values of 0.20, 0.35, 0.46, 0.81, 1.70, and 2.30. Lysochelin production in cultures at each of these OD_600_ values was analyzed as described above. For the growth assay of the Δ*lenB2* strain, the strain was cultured in 25 mL M813m-Fe medium, and OD_600_ values were measured every 12 h during the first 3 days of incubation (since WT reached its maximum OD_600_ value at 72 h) or 24 h (after 3 days of incubation) using the same spectrophotometer. The experiments were repeated three times with three technical replicates each.

### Preparation and structure determination of the differential compound

To obtain sufficient quantities of the differential compound for structural elucidation, large-scale fermentation of the WT strain was performed. Briefly, the strain was inoculated into 20 L of M813m medium supplemented with 1% (w/v) GlcNAc, and incubated at 30°C with shaking at 220 rpm for 72 h. Following centrifugation (8,000 rpm, 10 min), the fermentation broth was extracted twice with an equal volume of butanol (containing 1% trifluoroacetic acid), yielding 29 g of crude extract. This crude extract was fractionated by column chromatography using CHROMATOREX RP-18 resin (150 g, 20–45 μm, FUJI SILYSIA, Japan) with a stepwise gradient elution of methanol-water mixtures. Based on HPLC analysis, fractions enriched in the differential compound were combined and dried to obtain 105 mg of Fraction a (Fr. a). Subsequently, Fr. a was further purified by Sephadex™ LH-20 column chromatography (GE Healthcare, Sweden) eluted with methanol. HPLC monitoring led to the collection of Fr. a1 (19.5 mg), which was subjected to additional purification using a smaller RP-18 column (40 g). Finally, 2.5 mg of the pure differential compound was eluted with 20% (v/v) methanol. The purified compound was dissolved in deuterated methanol (methanol-*d*4), and ^1^H-NMR and ^13^C-NMR spectra were recorded on a Bruker AVANCE NEO 600 spectrometer operating at 600 MHz (^1^H) and 150 MHz (^13^C). The molecular weight of the differential compound was determined by LC-MS (Thermo, LCQ FLEET). The authentic *p*-hydroxybenzaldehyde (=99%GC, Aladdin, Shanghai, China) was purchased as a standard.

### RNA isolation and qRT-PCR

Total RNA was isolated from WT and its mutant derivatives cultured in M813m or M813m-Fe medium for 24 h, using the SPARKeasy Improved Bacteria RNA Isolation Kit (SparkJade) following the manufacturer’s instructions. The extracted RNA was reverse-transcribed into complementary DNA (cDNA) with the EasyScript One-Step gDNA Removal and cDNA Synthesis Supermix (Transgen) following the manufacturer’s instructions. Quantitative real-time PCR (qRT-PCR) was conducted on a LightCycler^®^ 96 System (Roche) with PerfectStart Green qPCR Supermix (Transgen) following the manufacturer’s instructions, using gene-specific primers listed in Supplementary Table S2. The qPCR conditions were as follows: initial denaturation at 94°C for 30 s, followed by 40 cycles of denaturation at 94°C for 5 s, annealing at 60°C for 15 s, and extension at 72°C for 10 s. The 16S rRNA gene was used as the internal reference gene. Relative transcriptional levels of target genes were normalized to the 16S rRNA gene and calculated using the 2^(-ΔΔCt) method (Livak and Schmittgen, 2001). Results were expressed as the fold change relative to the transcriptional level of each gene in the wild-type strain at the initial experimental time point. All data represent the mean values of three independent biological experiments, each with three technical replicates.

### Statistical analysis

Statistical analyses were conducted using one-way analysis of variance (ANOVA) followed by Tukey’s *post-hoc* test, with statistical significance defined as *p* < 0.05 or *p* < 0.01.

## Results

### GlcNAc induced the *p*-hydroxybenzaldehyde production in oligotrophic medium

**FIGURE 1:**
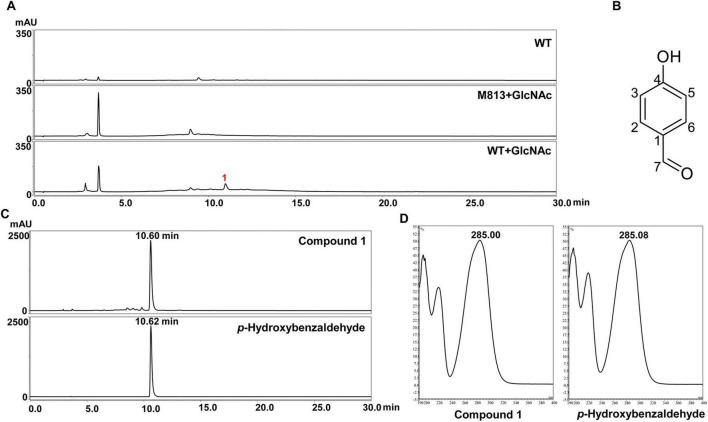
Induction of *p*-hydroxybenzaldehyde biosynthesis by GlcNAc. **(A)** HPLC analysis of the culture extracts from *Lysobacter* sp. 3655 grown in M813m medium (WT) or M813m with GlcNAc (WT + GlcNAc) for 72 h, the M813m with GlcNAc medium (M813 + GlcNAc) was used as the blank control. 1, Compound **1**. **(B)** Structure of *p*-hydroxybenzaldehyde. HPLC analysis of the retention time **(C)** and absorption spectrum **(D)** of Compound **1** and *p*-hydroxybenzaldehyde standard.

*Lysobacter* degrades cell wall of fungal to release chitin, and it was found that chitin significantly induced the production of antifungal HSAF (Yue et al., 2021). To further investigate the effect of chitin on the metabolites profile of environmental bacterial *Lysobacter*, we added chitin, chitosan oligosaccharide (the oligosaccharide hydrolysis product of chitin), and GlcNAc (the monomer of chitin) to different media for the cultivation of *Lysobacter* sp. 3655 (wild-type, WT), respectively. Crude extracts were analyzed by HPLC, and a unique compound (**1**) was found when WT was cultured in oligotrophic M813m medium supplemented with GlcNAc, compared with cultures grown in the same medium without GlcNAc (Figure 1A). As expected, the new peak was absent in sterile blank medium (Figure 1A), indicating that compound **1** was induced in in WT strain by GlcNAc. However, no significant differences in metabolite profiles were observed when WT was cultured in media supplemented with chitin or chitosan oligosaccharide (Supplementary Figure S1). Subsequently, large-scale fermentation of the WT strain was performed, and a pure sample (2.5 mg) was obtained via a series of column chromatography steps. The isolated compound was subjected to LC-MS and NMR analyses. LC-MS exhibited a molecular ion peak at m/z 121.03 [M-H]^–^ (Supplementary Figure S2). In the ^1^H-NMR spectrum (600 MHz, Methanol-*d*4), the compound exhibited chemical shifts at δ 9.77 (s, 1H, H-7), 7.79–7.77 (d, *J* = 8.6 Hz, 2H, H-2/6) and 6.93–6.91 (d, *J* = 8.6 Hz, 2H, H-3/5) (Supplementary Figure S3). The ^13^C-NMR spectrum showed carbon resonances at δ 192.84 (C-7), 165.20 (C-4), 133.44 (C-2/6), 130.33 (C-1) and 116.87 (C-3/5) (Supplementary Figure S4). Based on the combined data from LC-MS, ^1^H-NMR and ^13^C-NMR spectra, compound **1** was identified as *p*-hydroxybenzaldehyde (Figure 1B). To confirm this identification, authentic *p*-hydroxybenzaldehyde was purchased as a standard. The retention time and UV absorption spectrum of the purified compound were consistent with those of the authentic standard, further supporting the compound is *p*-hydroxybenzaldehyde (Figures 1C,D).

### Induction of *p*-hydroxybenzaldehyde was related to the utilization of GlcNAc and L-phenylalanine metabolism

**FIGURE 2:**
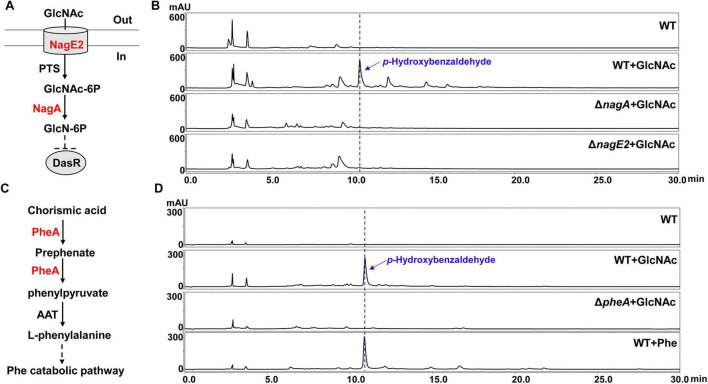
Effects of the GlcNAc utilization and L-phenylalanine metabolism on *p*-hydroxybenzaldehyde production. **(A)** The putative part of GlcNAc catabolism in *Lysobacter* sp. 3655. GlcNAc is taken up and phosphorylated by the permease NagE2 and the phosphotransferase system (PTS). The intracellular N-acetylglucosamine-6-phosphate (GlcNAc-6P) is then deacetylated by NagA, a GlcNAc-6P deacetylase, to form glucosamine-6-phosphate (GlcN-6P), which may serve as the allosteric effector of DasR, the regulator responsible for GlcNAc utilization. “Out” refers to the extracellular environment, and “In” refers to the intracellular environment. **(B)** HPLC analysis of *p*-hydroxybenzaldehyde production in WT, WT with GlcNAc (WT + GlcNAc) and the deletion mutants of GlcNAc utilization genes with GlcNAc (Δ*nagA* + GlcNAc, Δ*nagE2* + GlcNAc) grown in M813m medium for 72 h. **(C)** The putative biosynthetic pathway of L-phenylalanine in *Lysobacter* sp. 3655. Chorismic acid is converted to phenylpyruvic acid by a bifunctional chorismate mutase/prephenate dehydratase (PheA), and then aromatic-amino-acid transaminase (AAT) catalyzes phenylpyruvic acid to form L-phenylalanine which enters into its catabolic pathway. **(D)** HPLC analysis of *p*-hydroxybenzaldehyde production in WT, WT with GlcNAc (WT + GlcNAc), WT with L-phenylalanine (WT + Phe) and the deletion mutant of L-phenylalanine biosynthetic-related gene *pheA* with GlcNAc (Δ*pheA* + GlcNAc) grown in M813m medium for 72 h.

Several studies have documented that *p*-hydroxybenzaldehyde exhibits multiple pharmacological effects, covering anti- inflammatory, antioxidant, and vasodilatory activities (Bora et al., 2019; Ha et al., 2000; Xu et al., 2021). *P*-hydroxybenzaldehyde is found in various plants and algae, however, endogenous formation of *p*-hydroxybenzaldehyde in bacteria remains unreported. As mentioned above, GlcNAc stimulated *Lysobacter* sp. 3655 to generate *p*-hydroxybenzaldehyde. To further study the role of GlcNAc in inducing *p*-hydroxybenzaldehyde production, two GlcNAc utilization genes *nagA (orf143)* and *nagE2* (*orf5619*) were identified via homology-based searches (Supplementary Figures S5, S6). The gene *nagA* encodes an *N*-acetylglucosamine-6-phosphate deacetylase that catalyzes the conversion of *N*-acetylglucosamine-6-phosphate to glucosamine-6-phosphate; the gene *nagE2* encodes a permease of the phosphotransferase system for GlcNAc (Figure 2A; Craig et al., 2012). We constructed deletion mutants of these two genes (Δ*nagA* and Δ*nagE2*; Supplementary Figures S7, S8), and HPLC analysis revealed that neither Δ*nagA* nor Δ*nagE2* produced *p*-hydroxybenzaldehyde when cultured in GlcNAc-supplemented M813m medium (Figure 2B). This result indicates that *p*-hydroxybenzaldehyde biosynthesis is associated with GlcNAc utilization. Subsequently, we examined whether GlcNAc could induce the production of *p*-hydroxybenzaldehyde in different media. It was found that GlcNAc also promoted *Lysobacter* sp. 3655 to generate *p*-hydroxybenzaldehyde in YME rich medium and 1/10 TSB oligotrophic medium, though the yield of this compound was relatively lower in 1/10 TSB medium (Supplementary Figures S9, S10). It suggests that this mechanism exhibits a certain degree of conservation under different culture conditions.

In *Streptomyces*, GlcNAc modulates morphological differentiation and secondary metabolites biosynthesis via the GntR-family regulator DasR, which also directly regulates GlcNAc metabolism by controlling the GlcNAc regulon (Rigali et al., 2006). Based on this conserved mechanism, we hypothesized that a DasR homologue in *Lysobacter* sp. 3655 might mediate GlcNAc-induced *p*-hydroxybenzaldehyde production. A DasR-like protein-encoding gene (*dasR*, *orf145*) was identified via homology-based searches (Supplementary Figure S11), and the *dasR* deletion mutant (Δ*dasR*) was subsequently constructed (Supplementary Figure S12). However, GlcNAc-induced *p*-hydroxybenzaldehyde production in the Δ*dasR* strain was comparable to that in the WT strain (Supplementary Figure S13). These results indicate that GlcNAc-induced *p*-hydroxybenzaldehyde production is independent of the DasR-like protein in *Lysobacter* sp. 3655.

To our knowledge, the biosynthetic pathway of *p*-hydroxybenzaldehyde in microbes has not been reported, while *p*-hydroxybenzaldehyde derives from L-phenylalanine catabolism in some plants (Batista et al., 2018; Song T. et al., 2024). To explore the involvement of L-phenylalanine metabolism in *p*-hydroxybenzaldehyde biosynthesis, an L-phenylalanine biosynthesis-related gene *pheA* (*orf2468*) was identified through homology search in *Lysobacter* sp. 3655 (Supplementary Figure S14). This gene encodes a bifunctional chorismate mutase/prephenate dehydratase (*PheA*), which converts chorismic acid to phenylpyruvic acid (Figure 2C; Song T. et al., 2024). A deletion mutant of *pheA* (Δ*pheA*) was constructed (Supplementary Figure S15), and the results showed that Δ*pheA* completely abolished the *p*-hydroxybenzaldehyde production in GlcNAc-supplemented M813m medium (Figure 2D). This finding highlights the vital role of L-phenylalanine biosynthesis in *p*-hydroxybenzaldehyde generation. Furthermore, exogenous addition of L-phenylalanine induced *p*-hydroxybenzaldehyde production in *Lysobacter* sp. 3655 (Figure 2D), suggesting that *p*-hydroxybenzaldehyde biosynthesis is linked with L-phenylalanine catabolism.

### GlcNAc restored the lysochelin production in QS signal 4-HBA deficient strain

**FIGURE 3:**
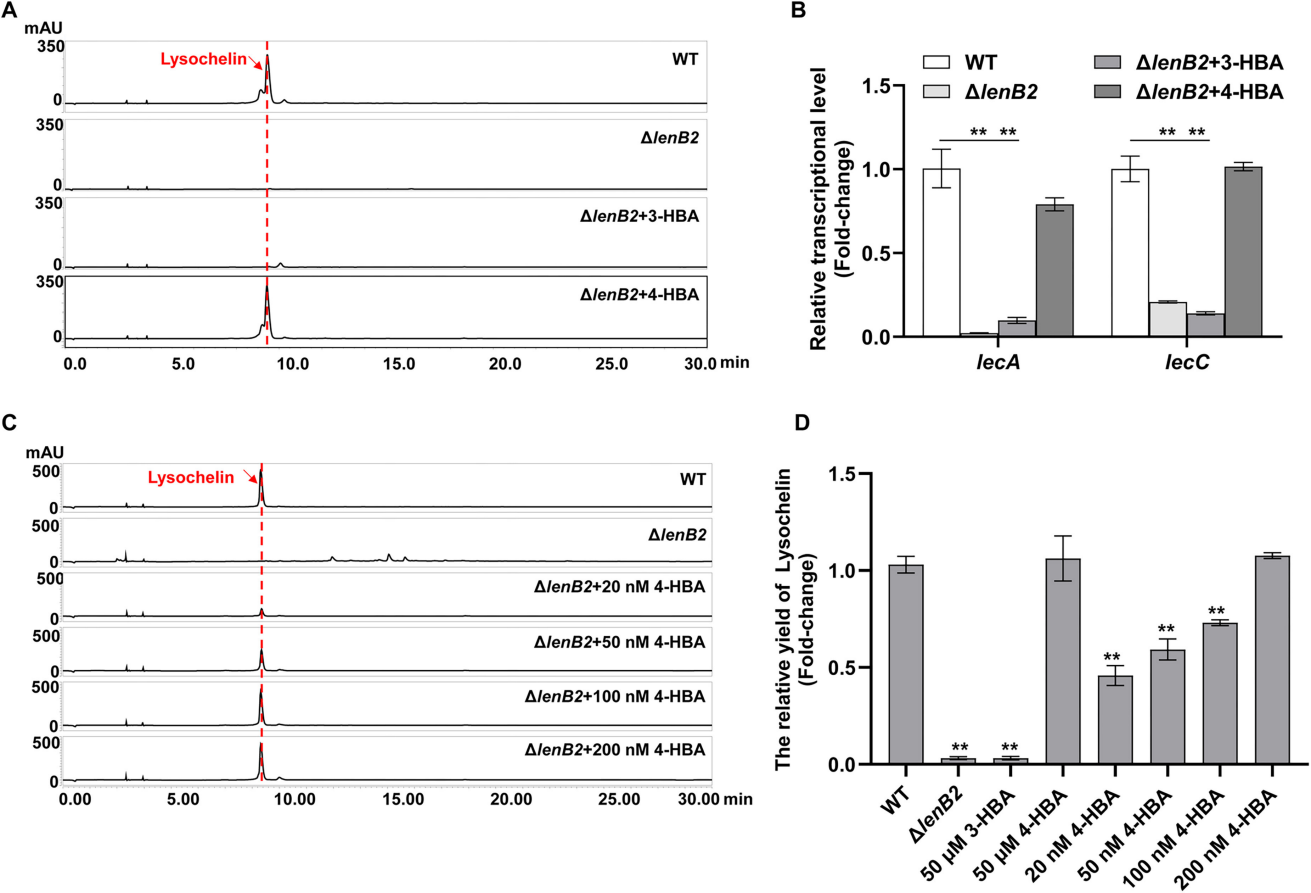
Regulation of the lysochelin production by quorum sensing. **(A)** HPLC analysis of lysochelin production in WT, the deletion mutant of the DF signal biosynthetic gene (Δ*lenB2*), and the Δ*lenB2* strain with 3-HBA or 4-HBA grown in M813m-Fe medium for 72 h. **(B)** Transcriptional analysis of the lysochelin biosynthetic genes (*lecA* and *lecC*) of WT (white columns), Δ*lenB2* (light gray columns), Δ*lenB2* with 3-HBA (gray columns) and Δ*lenB2* with 4-HBA (dark gray columns) grown in M813m-Fe medium for 24 h. **(C)** HPLC analysis of lysochelin production in the Δ*lenB2* strain grown in M813m-Fe medium containing different concentrations of 4-HBA (final concentrations of 0, 20, 50, 100, and 200 nM) for 72 h. **(D)** Quantification of lysochelin in the cultures from **(A,C)**. Data are presented as averages of three independent experiments, each conducted in triplicate. Statistical analyses were conducted using one-way analysis of variance (ANOVA) followed by Tukey’s *post-hoc* test. **p < 0.01.

In our previous work, we observed that the deletion of *lenB2* (*orf644*) significantly reduced lysochelin production in rich natural medium (Zhang et al., 2024). The gene *lenB2* encodes a pteridine-dependent dioxygenase-like protein and is involved in the biosynthesis of 3-hydroxybenzoic acid (3-HBA) and 4-HBA which are two QS signals in *Lysobacter* sp. 3655 (Zhang et al., 2024). As iron removal was not feasible in the rich natural medium, the iron-deficient oligotrophic synthetic medium (M813-Fe medium) was employed to cultivate the Δ*lenB2* strain for further investigation into the regulatory role of the QS system in lysochelin biosynthesis. Results showed that lysochelin was barely detectable in the Δ*lenB2* strain (Figures 3A,D). Previous study has revealed that the deletion of *lenB2* reduced the transcriptional levels of the lysochelin biosynthetic genes *lecA* (*orf2903*, encoding isochorismate synthase) and *lecC* (*orf2905*, encoding isochorimatase) in rich natural medium (Zhang et al., 2024). Similarly, the transcriptional levels of *lecA* and *lecC* were dramatically decreased in the Δ*lenB2* strain cultured on M813-Fe medium (Figure 3B). Additionally, the growth of the Δ*lenB2* strain was severely impaired, with cell density reduced to 23% of that in the WT strain (Supplementary Figure S16). As expected, exogenous addition of 4-HBA restored both growth and lysochelin production in the Δ*lenB2* strain (Figures 3A,D and Supplementary Figure S16). Consistently, 4-HBA significantly upregulated the transcriptional levels of *lecA* and *lecC* in the mutant (Figure 3B). In contrast, 3-HBA did not affect cell density or lysochelin biosynthesis in the Δ*lenB2* strain (Figure 3 and Supplementary Figure S16). We then examined the effect of 4-HBA concentration on lysochelin production and growth of the Δ*lenB2*strain. The results showed that 20 nM 4-HBA significantly enhanced cell density and lysochelin production, and both parameters were restored to WT levels when the 4-HBA concentration reached 200 nM (Figures 3C,D and Supplementary Figure S17). These findings demonstrate that the QS signal 4-HBA promotes lysochelin biosynthesis in *Lysobacter* sp. 3655.

The Δ*lenB2* strain exhibited a severe growth defect; its OD_600_ value remained at 0.3∼0.4 even after 10 d of incubation in M813m-Fe medium (Supplementary Figure S18). Given that cell density is associated with siderophore biosynthesis (Darch et al., 2012), we investigated the relationship between the cell density of WT and lysochelin production. The results showed that the WT did not produce lysochelin at an OD_600_ below 0.20 (Supplementary Figure S19). It initiated lysochelin biosynthesis in the early exponential growth phase (OD_600_ = 0.35), reached maximal production at the mid-exponential phase (OD_600_ = 0.81), and exhibited decreased production at the late exponential phase (OD_600_ = 1.70, OD_600_ = 2.30) (Supplementary Figure S19). Then we compared lysochelin production between WT and Δ*lenB2* at a consistent OD_600_ value of 0.35. As expected, a lysochelin-specific signal peak was observed in WT, whereas no corresponding peak was present in the Δ*lenB2* strain (Supplementary Figure S20). This indicated that the regulation of lysochelin biosynthesis by 4-HBA is related to the absence of the signaling molecule rather than independence from cell density.

**FIGURE 4:**
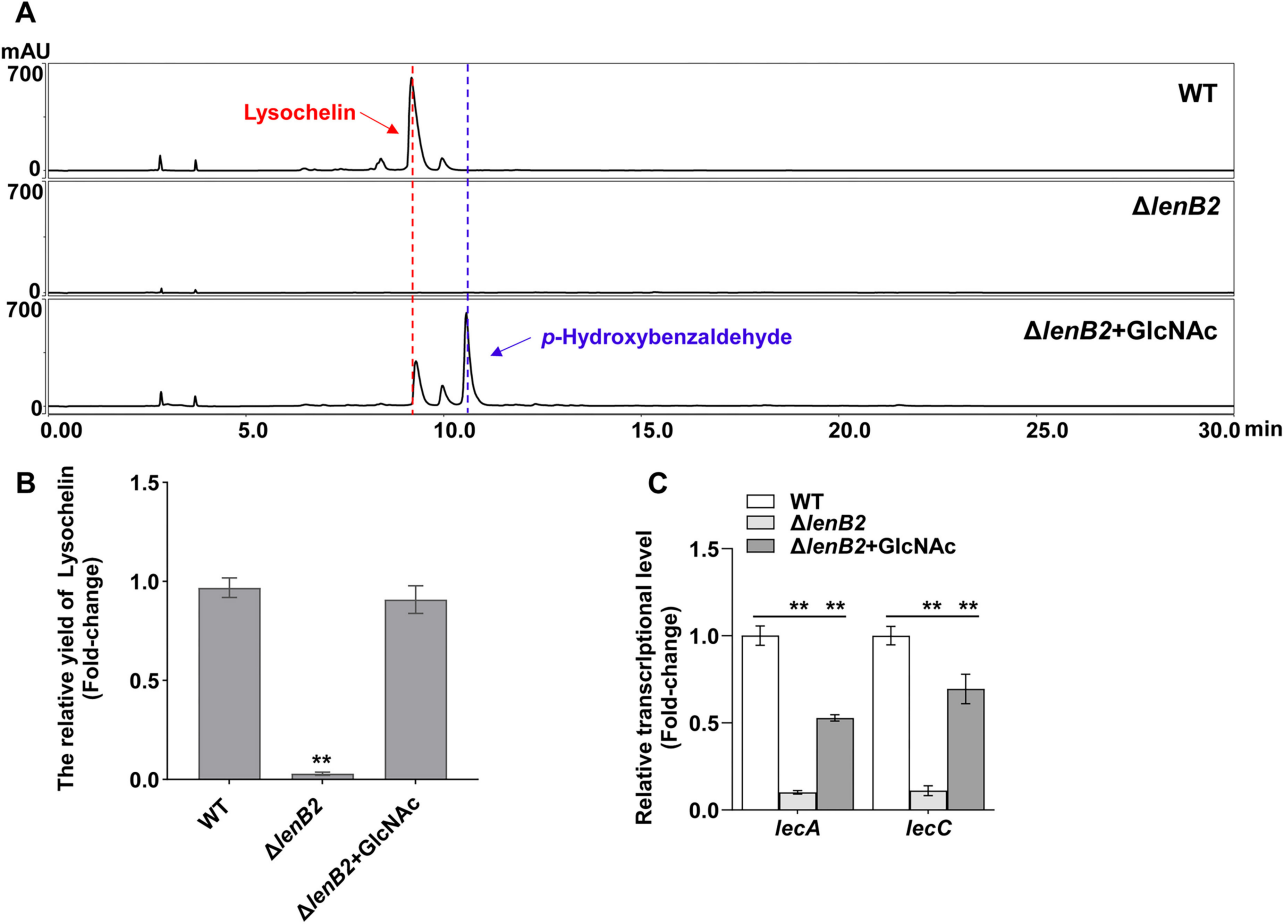
Recovery of lysochelin biosynthesis in the Δ*lenB2* strain by GlcNAc. **(A)** HPLC analysis of lysochelin production in WT, Δ*lenB2* and Δ*lenB2* with GlcNAc grown in M813m-Fe medium for 72 h. **(B)** Quantification of lysochelin in the cultures from **(A)**. **(C)** Transcriptional analysis of the lysochelin biosynthetic genes (*lecA* and *lecC*) of WT (white columns), Δ*lenB2* (light gray columns) and Δ*lenB2* with GlcNAc (gray columns) grown in M813m-Fe medium for 24 h. Data are presented as averages of three independent experiments, each conducted in triplicate. Statistical analyses were conducted using one-way analysis of variance (ANOVA) followed by Tukey’s *post-hoc* test. **p < 0.01.

**FIGURE 5:**
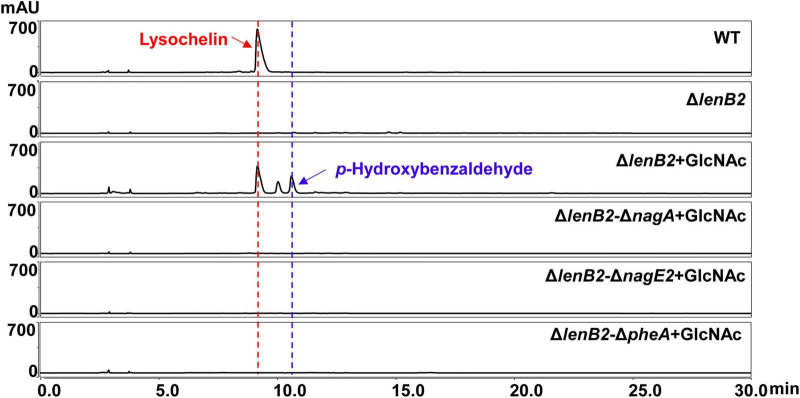
Effects of the GlcNAc utilization and L-phenylalanine metabolism on lysochelin production. HPLC analysis of lysochelin production in WT, Δ*lenB2*, Δ*lenB2* with GlcNAc (Δ*lenB2* + GlcNAc), Δ*lenB2*-Δ*nagA* with GlcNAc (Δ*lenB2*-Δ*nagA* + GlcNAc), Δ*lenB2*-Δ*nagE2* with GlcNAc (Δ*lenB2*-Δ*nagE2* + GlcNAc) and Δ*lenB2*-Δ*pheA* with GlcNAc (Δ*lenB2*-Δ*pheA* + GlcNAc) grown in M813m-Fe medium for 72 h.

Surprisingly, exogenous GlcNAc supplementation restored lysochelin production in the Δ*lenB2* strain to WT levels and significantly increased cell density (Figures 4A,B and Supplementary Figure S21). Consistent with this observation, GlcNAc addition markedly upregulated the transcriptional levels of *lecA* and *lecC* in the Δ*lenB2* strain (Figure 4C). Interestingly, GlcNAc induced the *p*-hydroxybenzaldehyde biosynthesis in the Δ*lenB2* strain as well (Figure 4A). However, GlcNAc did not affect lysochelin production in the WT strain, whereas *p*-hydroxybenzaldehyde was produced as expected (Supplementary Figure S22). In order to confirm the complementary effect of GlcNAc, we deleted *nagA* (*orf143*) or *nagE2* (*orf5619*) in the Δ*lenB2* background, generating two double mutants Δ*lenB2*-Δ*nagA* and Δ*lenB2*-Δ*nagE2* (Supplementary Figures S23, S24). HPLC analysis revealed that deletion of either *nagA* (*orf143*) or *nagE2* (*orf5619*) abolished GlcNAc-induced lysochelin production (Figure 5). Furthermore, exogenous GlcNAc could not restore the cell density of the Δ*lenB2*-Δ*nagA* and Δ*lenB2*-Δ*nagE2* double mutants, whose cell density remained similar to that of the Δ*lenB2* strain grown without GlcNAc supplementation (Supplementary Figure S25). These results indicated that GlcNAc catabolism is required for the GlcNAc-dependent restoration of lysochelin production and growth of the Δ*lenB2* strain in M813-Fe medium. However, the deletion of *dasR* (*orf145*) ether in WT or Δ*lenB2* did not affect lysochelin yield (Supplementary Figures S26, S27), indicating that the regulation of lysochelin biosynthesis by GlcNAc might be independent of DasR-like regulator in *Lysobacter* sp. 3655. Furthermore, DasR had no influence on *p*-hydroxybenzaldehyde production in the Δ*lenB2* strain, which is consistent with the phenomenon observed in the WT strain (Figure 5 and Supplementary Figure S13).

### GlcNAc regulated the lysochelin production through the induction of *p*-hydroxybenzaldehyde biosynthesis

GlcNAc could activate *p*-hydroxybenzaldehyde production in the WT strain grown in M813m medium (Figure 1A), and we also observed that GlcNAc induced both *p*-hydroxybenzaldehyde and lysochelin production in the Δ*lenB2* strain cultured in M813m-Fe medium, indicating these two compounds were produced synchronously (Figure 4A). To test whether GlcNAc-induced *p*-hydroxybenzaldehyde induction is involved in lysochelin biosynthesis, we first evaluated different GlcNAc concentrations (final concentrations of 0, 0.25, 0.5, 1, and 2%, w/v) for their effects on *p*-hydroxybenzaldehyde and lysochelin production. Results showed that both *p*-hydroxybenzaldehyde and lysochelin production were dramatically induced by the addition of 0.25% GlcNAc to M813m-Fe medium, with yields slightly increasing when the GlcNAc concentration reached 1% (Supplementary Figure S28).

**FIGURE 6:**
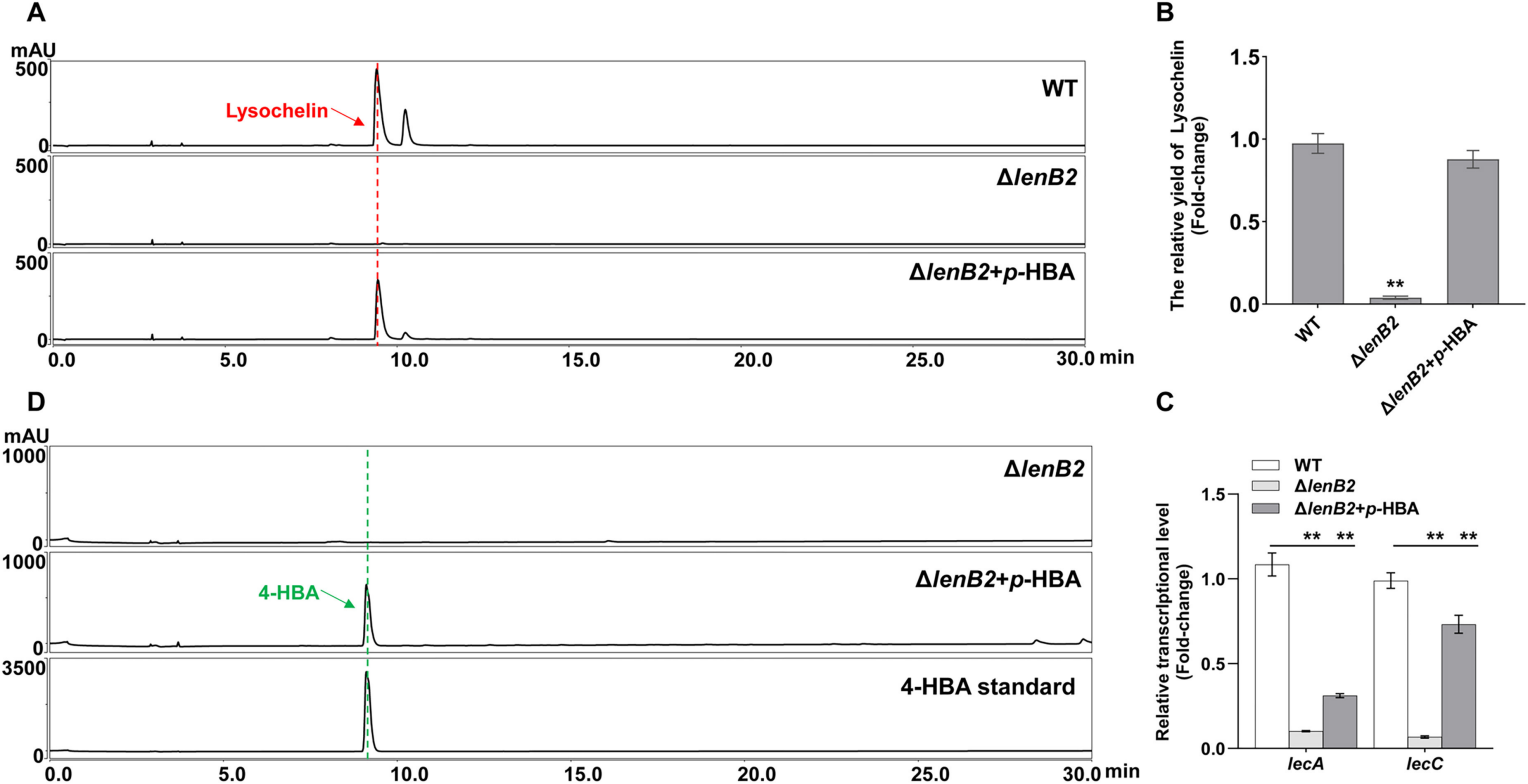
Restoration of lysochelin biosynthesis in the Δ*lenB2* strain by *p*-hydroxybenzaldehyde. **(A)** HPLC analysis of lysochelin production in WT, Δ*lenB2* and Δ*lenB2* with *p*-hydroxybenzaldehyde (*p*-HBA) grown in M813m-Fe medium for 72 h. **(B)** Quantification of lysochelin in the cultures from **(A)**. **(C)** Transcriptional analysis of the lysochelin biosynthetic genes (*lecA* and *lecC*) of WT (white columns), Δ*lenB2* (light gray columns) and Δ*lenB2* with *p*-HBA (gray columns) grown in M813m-Fe medium for 24 h. **(D)** HPLC analysis of 4-HBA production in Δ*lenB2*, Δ*lenB2* with *p*-HBA grown in M813m medium for 72 h, pure 4-HBA was used as standard. Data are presented as averages of three independent experiments, each conducted in triplicate. Statistical analyses were conducted using one-way analysis of variance (ANOVA) followed by Tukey’s *post-hoc* test. **p < 0.01.

Since deletion of *pheA* (*orf2468*) in the WT strain abolished *p*-hydroxybenzaldehyde production, we constructed a Δ*lenB2*-Δ*pheA* double mutant by deleting *pheA* (*orf2468*) in the Δ*lenB2* background (Supplementary Figure S29). Neither *p*-hydroxybenzaldehyde nor lysochelin was detected in the Δ*lenB2*-Δ*pheA* strain supplemented with GlcNAc (Figure 5). Furthermore, the Δ*lenB2*-Δ*nagA* and Δ*lenB2*-Δ*nagE2* double mutants were also unable to produce *p*-hydroxybenzaldehyde and lysochelin upon GlcNAc supplementation (Figure 5). Collectively, these results suggest that *p*-hydroxybenzaldehyde might contribute to lysochelin production in the Δ*lenB2* strain. To test this hypothesis, we added *p*-hydroxybenzaldehyde to the Δ*lenB2* strain cultured in M813m-Fe medium. The results showed that *p*-hydroxybenzaldehyde clearly restored lysochelin production to WT levels and dramatically enhanced the transcriptional levels of *lecA* and *lecC* in the Δ*lenB2* strain (Figures 6A–C). Moreover, *p*-hydroxybenzaldehyde restored the cell density of the Δ*lenB2* strain (Supplementary Figure S30). It appeared that *p*-hydroxybenzaldehyde and 4-HBA exerted the similar effect on the growth and lysochelin biosynthesis of the Δ*lenB2* strain. In plants, *p*-hydroxybenzaldehyde is converted to 4-HBA by 4-hydroxybenzaldehyde dehydrogenase (Song T. et al., 2024; Schnitzler et al., 1992), thus we speculated that *p*-hydroxybenzaldehyde could be converted to 4-HBA in the Δ*lenB2* strain. To verify it, we added *p*-hydroxybenzaldehyde to the 4-HBA-deficient strain Δ*lenB2* cultured in M813m medium, and a large amount of 4-HBA was detected (Figure 6D). Thus, *p*-hydroxybenzaldehyde restores lysochelin production in the Δ*lenB2* strain by participating in the production of 4-HBA. However, L-phenylalanine failed to restore lysochelin production of the Δ*lenB2* strain cultured in M813m-Fe medium, a phenomenon likely attributable to the low yield of *p*-hydroxybenzaldehyde elicited by L-phenylalanine in the Δ*lenB2* strain (Supplementary Figure S31).

### The intermediates from L-phenylalanine catabolism restored lysochelin production in the Δ*lenB2* strain

**FIGURE 7:**
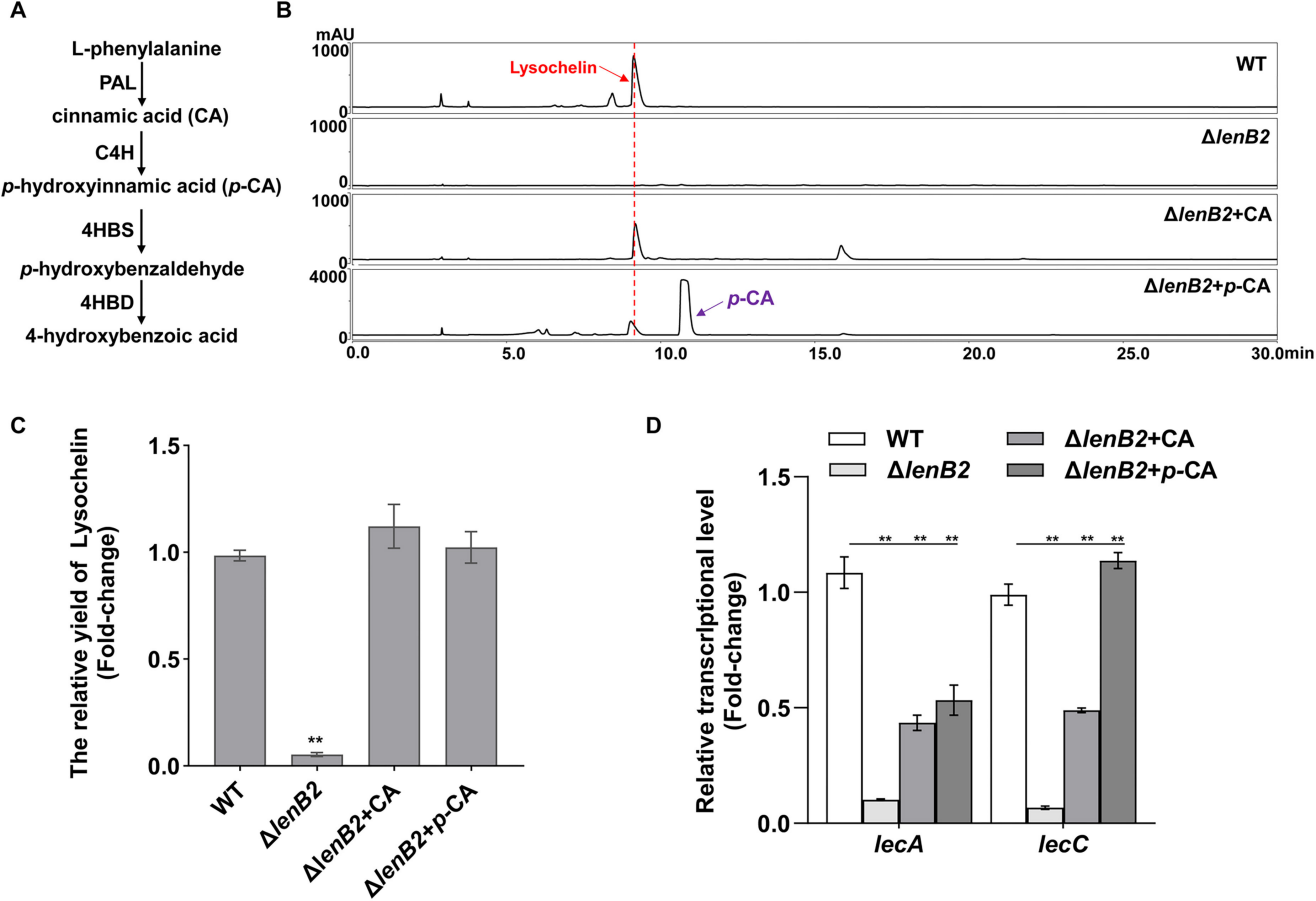
Recovery of lysochelin biosynthesis in the Δ*lenB2* strain by L-phenylalanine intermediates. **(A)** The putative L-phenylalanine catabolism pathway in plants (Song T. et al., 2024). L-phenylalanine is first converted to cinnamic acid by L-phenylalanine ammonia-lyase (PAL). Cinnamic acid is then hydroxylated by cinnamate 4-hydroxylase (C4H) to form *p*-hydroxycinnamic acid, which is further transformed into *p*-hydroxybenzaldehyde via 4-hydroxybenzaldehyde synthase (4HBS). In the final step, *p*-hydroxybenzaldehyde is oxidized to 4-HBA by 4-hydroxybenzaldehyde dehydrogenase (4HBD). **(B)** HPLC analysis of lysochelin production in WT, Δ*lenB2*, Δ*lenB2* with cinnamic acid (CA) and Δ*lenB2* with *p*-hydroxy cinnamic acid (*p*-CA) grown in M813m-Fe medium for 72 h. **(C)** Quantification of lysochelin in the cultures from **(A)**. **(D)** Transcriptional analysis of the lysochelin biosynthetic genes (*lecA* and *lecC*) of WT (white columns), Δ*lenB2* (light gray columns), Δ*lenB2* with CA (gray columns) and Δ*lenB2* with *p*-CA (dark gray columns) grown in M813m-Fe medium for 24 h. Data are presented as averages of three independent experiments, each conducted in triplicate. Statistical analyses were conducted using one-way analysis of variance (ANOVA) followed by Tukey’s *post-hoc* test. **p < 0.01.

In plants, *p*-hydroxybenzaldehyde is one of the intermediates of L-phenylalanine catabolism (Song T. et al., 2024). The catabolic pathway proceeds as follows: L-phenylalanine is first degraded to cinnamic acid by L-phenylalanine ammonia-lyase (PAL); cinnamic acid is then hydroxylated to *p*-hydroxycinnamic acid by cinnamic acid-4-hydroxylase (C4H); *p*-hydroxycinnamic acid is subsequently converted to *p*-hydroxybenzaldehyde by 4-hydroxybenzaldehyde synthase (4HBS); finally, *p*-hydroxybenzaldehyde is oxidized to 4-HBA by 4-hydroxybenzaldehyde dehydrogenase (Figure 7A; Song T. et al., 2024). Given that no relevant literature has reported the biosynthetic pathway of *p*-hydroxybenzaldehyde in microorganisms, we used the biosynthetic route of *p*-hydroxybenzaldehyde identified in plants as a conceptual reference.

Since both cinnamic acid (CA) and *p*-hydroxycinnamic acid (*p*-CA) are precursors of *p*-hydroxybenzaldehyde, these two intermediates were exogenously added to the Δ*lenB2* strain to assess their effects on lysochelin production. It was found that cinnamic acid and *p*-hydroxycinnamic acid could restore lysochelin production and cell density in the Δ*lenB2* strain, as well as upregulate the transcriptional levels of *lecA* and *lecC* (Figures 7B–D and Supplementary Figure S32). However, no *p*-hydroxybenzaldehyde or 4-HBA was detected in the Δ*lenB2* strain following exogenous addition of cinnamic acid or *p*-hydroxycinnamic acid to M813m medium (Supplementary Figure S33). These results indicate that the two L-phenylalanine catabolic intermediates (cinnamic acid and *p*-hydroxycinnamic acid) contribute to lysochelin biosynthesis via 4-HBA-independent pathway.

**FIGURE 8:**
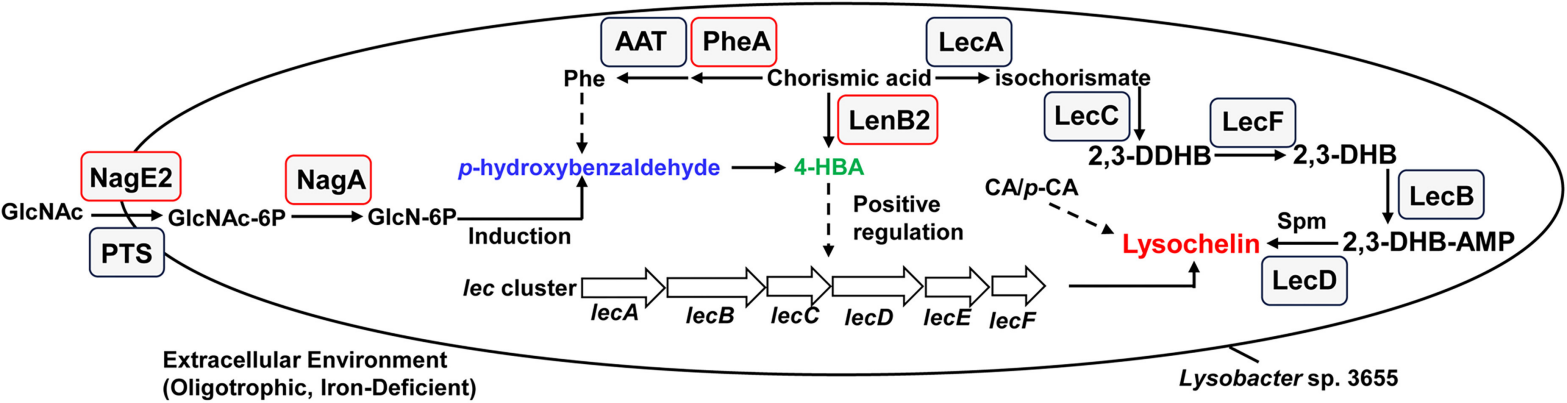
The possible mechanism of GlcNAc regulating siderophore lysochelin biosynthesis in *Lysobacter* sp. 3655. The lysochelin biosynthetic pathway proceeds as follows: chorismic acid is first catalyzed to isochorismate by isochorismate synthase (LecA); isochorismate is then converted to 2,3-Dihydro-2,3-dihydroxybenzoate (2, 3-DDHB) by isochorimatase (LecC); 2, 3-DDHB is subsequently dehydrogenated to 2,3-Dihydroxybenzoate (2, 3-DHB) by 2,3-dihydro-2,3-DHBA dehydrogenase (LecF); then 2,3-DHB-AMP ligase (LecB) activates the carboxylate of 2,3-DHB as an adenylate (2,3-DHB-AMP); finally, *lecD*-encoded condensation enzyme catalyzes spermidine (Spm) and two units of 2,3-DHB-AMP to form lysochelin (Miller et al., 2023). Under iron-deficient oligotrophic conditions, GlcNAc is transported and converted to glucosamine-6-phosphate (GlcN-6P) by NagE2, PTS system and NagA. Then GlcN-6P promotes *p*-hydroxybenzaldehyde production, which may occur through L-phenylalanine metabolism. Subsequently, *p*-hydroxybenzaldehyde is converted to 4-HBA, which could also be generated from chorismic acid catalyzed by a pteridine-dependent dioxygenase (LenB2). Lastly, 4-HBA regulates lysochelin biosynthesis through enhancing the expression of its biosynthetic gene cluster (*lec* cluster). Moreover, cinnamic acid (CA)/*p*-hydroxycinnamic acid (*p*-CA) restored the lysochelin production via 4-HBA-independent pathway. The research objects in this work are labeled using red boxes, the dashed arrows indicate uncharacterized pathways.

Taken together, we propose a possible pathway through which GlcNAc may regulate lysochelin biosynthesis in *Lysobacter* sp. 3655 (Figure 8). In iron-deficient oligotrophic environments, GlcNAc is taken up and transformed into glucosamine-6-phosphate (GlcN-6P) by NagE2, the PTS system, and NagA. GlcN-6P then stimulates the production of *p*-hydroxybenzaldehyde, which is likely mediated through L-phenylalanine metabolism. *P*-hydroxybenzaldehyde is further converted to 4-HBA via a potential pathway; this compound can also be biosynthesized from chorismic acid via catalysis by a pteridine-dependent dioxygenase (LenB2). Ultimately, 4-HBA controls lysochelin biosynthesis by upregulating the expression of its biosynthetic gene cluster (*lec* cluster). Moreover, CA/*p*-CA promoted the lysochelin production via 4-HBA-independent pathway.

## Discussion

In this work, we found that GlcNAc significantly induces the production of a differential compound in *Lysobacter* sp. 3655 grown in the oligotrophic M813m medium. Combined NMR and MS analyses identified this compound as *p*-hydroxybenzaldehyde. This is the first report describing the isolation of *p*-hydroxybenzaldehyde from microorganisms under oligotrophic medium without the addition of its relevant precursors (e.g., cresol, lignin and *p*-coumarate). Oligotrophy is a typical condition in the native habitats of soil-derived *Lysobacter* sp. 3655, and it indicates that the strain could respond to a specific carbon source (GlcNAc) and biosynthesizing the aromatic metabolite. Subsequently, we constructed deletion mutants of the GlcNAc catabolic gene *nagA* (*orf143*) and transport gene *nagE2* (*orf5619*). Both mutants completely abolished GlcNAc-induced *p*-hydroxybenzaldehyde production. However, this process is independent of DasR regulator which controls GlcNAc-induced morphological differentiation and secondary metabolites biosynthesis in *Streptomyces* (Rigali et al., 2006). *Streptomyces* and *Lysobacter* are phylogenetically very distant and belong to two distinct bacterial phyla, it is probably that DasR-like protein (ORF145) did not regulate *p*-hydroxybenzaldehyde production in *Lysobacter* sp. 3655. Moreover, it indicates that novel regulators in *Lysobacter* sp. 3655 govern the metabolic changes triggered by GlcNAc.

Microorganisms possess an excellent ability to degrade various aromatic compounds (e.g., cresol, lignin and *p*-coumarate), yielding *p*-hydroxybenzaldehyde as an intermediate metabolite (Londry et al., 1999; Ali et al., 2024; Hirakawa et al., 2012). To our knowledge, no studies have addressed the endogenous biosynthetic pathway of *p*-hydroxybenzaldehyde in microorganisms, whereas *p*-hydroxybenzaldehyde is derived from the L-phenylalanine catabolic pathway in some plants (Batista et al., 2018; Song T. et al., 2024). However, no homologous genes encoding PAL, C4H, or 4HBS were identified in *Lysobacter* sp. 3655. Moreover, exogenous addition of L-phenylalanine to the WT strain significantly induced *p*-hydroxybenzaldehyde production in M813m medium. This may suggest that the L-phenylalanine catabolic pathway in *Lysobacter* sp. 3655 differs from that in plants, despite both producing *p*-hydroxybenzaldehyde. L-phenylalanine originates from chorismic acid which is first converted to phenypyruvic acid by chorismite mutase and prephenate dehydratase, followed by the transamination of phenypyruvic acid (Song T. et al., 2024). The deletion of *pheA* (*orf2468*) completely abolished GlcNAc-induced *p*-hydroxybenzaldehyde production. These results suggest that *p*-hydroxybenzaldehyde production is connected with L-phenylalanine biosynthesis in *Lysobacter* sp. 3655. It is worth noting that *p*-hydroxybenzaldehyde may function as an intermediate that converting to 4-HBA or other metabolites in *Lysobacter* sp. 3655. *Pycnoporus cinnabarinus* accumulates a large amount of *p*-hydroxybenzaldehyde by converting *p*-coumaric acid in rich medium (Alvarado et al., 2001). However, we observed that *Lysobacter* sp. 3655 was able to utilize *p*-coumaric acid in oligotrophic medium, but no formation of *p*-hydroxybenzaldehyde was detected. It indicates that the conversion pathways of *p*-coumaric acid in *Pycnoporus cinnabarinus* and *Lysobacter* sp. 3655 are distinct. Moreover, GlcNAc stimulates *Lysobacter* sp. 3655 to produce *p*-hydroxybenzaldehyde in both YME rich medium and 1/10 TSB oligotrophic medium, suggesting that the induction mechanism is functional under diverse culture conditions. Together, two possible mechanisms are proposed for the GlcNAc-induced biosynthesis of *p*-hydroxybenzaldehyde: (i) GlcNAc may affect the global metabolic pathways, thereby redirecting some metabolites toward the biosynthesis of *p*-hydroxybenzoic acid, and (ii) GlcNAc may cause the accumulation of *p*-hydroxybenzaldehyde by either enhancing its upstream biosynthetic pathway or attenuating its downstream utilization pathway.

The deletion of 4-HBA biosynthetic gene *orf644* abolished lysochelin production in iron-deficient oligotrophic medium, suggesting that quorum sensing positively regulates siderophore biosynthesis in *Lysobacter* sp. 3655. It is well-documented that quorum sensing modulates siderophore production in microorganisms (Stintzi et al., 1998; Song Y. et al., 2024; Ren et al., 2005; Batista et al., 2024; McRose et al., 2018; Heffernan et al., 2024). In our study, we found that GlcNAc restored lysochelin production in Δ*lenB2*, implying GlcNAc controls lysochelin biosynthesis through quorum sensing in *Lysobacter* sp. 3655. To date, only one study has reported the regulation of siderophore production by GlcNAc (Craig et al., 2012). In *S. coelicolor*, GlcNAc relieves the repression of DasR on the expression of the iron utilization repressor gene *dmdR1*, and then the accumulated DmdR1 protein directly binds to the promoter region of the siderophore biosynthesis gene cluster, thereby inhibiting the production of the siderophores coelichelin and desferrioxamine (Craig et al., 2012). However, similar to *p*-hydroxybenzaldehyde, the GlcNAc-induced lysochelin production is independent of the DasR-like regulator in *Lysobacter* sp. 3655.

We found that exogenously added 4-HBA could restore lysochelin production in the Δ*lenB2* strain. Furthermore, exogenously supplied *p*-hydroxybenzaldehyde was converted to 4-HBA in the Δ*lenB2* strain. Therefore, we speculate that GlcNAc initially triggers the biosynthesis of *p*-hydroxybenzaldehyde in the Δ*lenB2* strain; this product is then transformed into 4-HBA, which ultimately mediates the restoration of lysochelin production in the Δ*lenB2* strain. These results suggest that the existence of multiple independent 4-HBA biosynthetic pathways in *Lysobacter* sp. 3655. Notably, 4-HBA was undetectable in the Δ*lenB2* strain, implying that LenB2 (ORF644) might serve as the sole source of 4-HBA under oligotrophic medium. Conversely, we propose that when LenB2 (ORF644) is inactivated, *Lysobacte*r sp. 3655 employs an alternative 4-HBA biosynthetic pathway derived from L-phenylalanine metabolism, and that GlcNAc may stimulate metabolic flux through the route to promote 4-HBA production. However, we identified only one medium that supports lysochelin production, as a suitable iron-deficient synthetic medium for the normal growth of *Lysobacter* sp. 3655 remains difficult to obtain. Nevertheless, we hypothesize that the GlcNAc-restored lysochelin production in a 4-HBA-deficient background is broadly functional under iron-deficient conditions. Moreover, the conversion of *p*-hydroxybenzaldehyde to 4-HBA requires further investigation since no typical 4-hydroxybenzaldehyde dehydrogenase-encoding gene was identified in *Lysobacter* sp. 3655 via homology-based searching. In our previous work, we found that maltose promotes the high-density cell growth of *Lysobacter* sp. 3655 and then creates an iron-deficient environment for lysochelin production (Zhang et al., 2024). However, iron-deficient oligotrophic medium was used for lysochelin biosynthesis in this study. Both converge on *lenB2*/4-HBA-dependent quorum sensing as a central regulatory node for lysochelin biosynthesis, confirming this QS system is involved in lysochelin production regardless of culture conditions. Together, they prove that lysochelin biosynthesis is controlled not only by iron limitation but also by specific carbon-source signals (maltose/GlcNAc), which integrate nutritional status, cell density, and secondary metabolism.

The rhizosphere is the natural habitat of *Lysobacter*, and it secretes a variety of hydrolytic enzymes (including chitinases, peptidases, proteases and cellulases) and bioactive secondary metabolites to lyse or kill diverse microorganisms as a nutrient source (Seccareccia et al., 2015). As a result, *Lysobacter* is widely employed in biological control. For instance, *Lysobacter antibioticus* protects *Oryza sativa* L. from *Xanthomonas oryzae* pv. oryzae infection; *Lysobacter capsci* controls Grapevine downy mildew of *Vitis vinifera* L.; and *Lysobacter enzymogenes* is effective in suppressing *Pythium aphanidermatum* infection in *Cucumis sativa* L. (Ji et al., 2008; Puopolo et al., 2014; Folman et al., 2004). Therefore, *Lysobacter* may interacts with other microorganisms in its niche. It is possible that when encountering other microorganisms, *Lysobacter* first degrades their cell walls using hydrolytic enzymes; consequently, large amounts of GlcNAc are released into the environment. Subsequently, GlcNAc is transported into *Lysobacter* and converted to GlcN-6P by NagE2, NagA and other proteins. Through an uncharacterized pathway, GlcN-6P induces *Lysobacter* to produce *p*-hydroxybenzaldehyde, which is further converted to 4-HBA. In turn, 4-HBA promotes biosynthesis of the siderophore lysochelin, thereby supporting improved growth of *Lysobacter* under iron-deficient environments. Thus, GlcNAc-driven regulation of lysochelin biosynthesis may contribute to improving the competitive capacity of *Lysobacter* in natural environments.

In summary, we found that GlcNAc induces *p*-hydroxybenza- ldehyde production, which might be associated with GlcNAc utilization and L-phenylalanine metabolism. Furthermore, 4-HBA positively regulates lysochelin biosynthesis in *Lysobacter* sp. 3655. GlcNAc or *p*-hydroxybenzaldehyde restores lysochelin production in the Δ*lenB2*strain by promoting the conversion of *p*-hydroxybenzaldehyde to 4-HBA, whereas cinnamic acid and *p*-hydroxycinnamic acid rescue lysochelin production via a 4-HBA-independent mechanism. This study provides new insights into the regulation of siderophore biosynthesis, offering valuable clues for investigating the biocontrol mechanisms in *Lysobacter*.

## Data Availability

The datasets presented in this study can be found in online repositories. The names of the repository/repositories and accession number(s) can be found in the article/Supplementary material.
