# Supplementary material for: N-acetylglucosamine regulates the siderophore lysochelin production through inducing the p-hydroxybenzaldehyde biosynthesis in Lysobacter sp. 3655
Source: Front Microbiol. 2026 Apr 24;17:1808803. doi: 10.3389/fmicb.2026.1808803 (PMC13153030; doi:10.3389/fmicb.2026.1808803)
Supplement: Supplementary file 1 [file Supplementary_file_1.docx]

***N*-acetylglucosamine regulates the siderophore lysochelin production through inducing the** ***p*-hydroxybenzaldehyde biosynthesis in *Lysobacter* sp. 3655**

Lin Jiang^†^, Xiaofang Yan^†^, Fang Zhang, Ailing Chen, Yixuan Chen, Yongbiao Zheng^*^, Lingjun Yu^*^

School of Life Sciences, Fujian Normal University, Fuzhou, Fujian, China

***Corresponding:**

Yongbiao Zheng

[yongbiaozheng@fjnu.edu.cn](mailto:yongbiaozheng@fjnu.edu.cn)

Lingjun Yu

[yulj113@fjnu.edu.cn](mailto:yulj113@fjnu.edu.cn)

^†^These authors contribute equally to this work.

**Table S1.** Bacterial strains and plasmids used in this study

| Bacterial strains/plasmids | Relevant characteristics*^a^* | Source/references |
| --- | --- | --- |
| *Lysobacter* sp. 3655 | Wild-type, Km^r^ | DSMZ |
| Δ*lenB2* | The deletion mutant of *lenB2* (*orf644*) in 3655, the same as ΔORF644 in the reference | (Zhang et al., 2024) |
| Δ*nagA* | The deletion mutant of *nagA* (*orf143*) in 3655 | This study |
| Δ*lenB2*-Δ*nagA* | The deletion mutant of *nagA* in Δ*lenB2* | This study |
| Δ*dasR* | The deletion mutant of *dasR* (*orf145*) in 3655 | This study |
| Δ*lenB2*-Δ*dasR* | The deletion mutant of *dasR* in Δ*lenB2* | This study |
| Δ*pheA* | The deletion mutant of *pheA* (*orf2468*) in 3655 | This study |
| Δ*lenB2*-Δ*pheA* | The deletion mutant of *pheA* in Δ*lenB2* | This study |
| Δ*nagE2* | The deletion mutant of *nagE2* (*orf5619*) in 3655 | This study |
| Δ*lenB2*-Δ*nagE2* | The deletion mutant of *nagE2* in Δ*lenB2* | This study |
| **Other bacteria** |  |  |
| *Escherichia coli* DH5α | Host strain for molecular cloning, F-φ80 *lac*ZΔM15 Δ(*lac*ZYA-*arg*F) U169 *end*A1 *rec*A1 *hsd*R17(rK⁻ mK⁺) *sup*E44 λ- *thi*-1 *gyr*A96 *rel*A1 *pho*A | Transgen |
| *E. coli* S17-1 | Strain for conjugation with *Lysobacter*, [C600::RP4-2 (Tc::Mu) (Km::Tn7)] *thi*-1 *pro*-82 *hsd*R17 (rK⁻ mK⁺) *rec*A1 *end*A1 *thi*E1 *cre*C510, Tpᵣ Smᵣ | Laboratory collection |
| **Plasmids** |  |  |
| pJQ200SK | Suicide vector, Gm^r^, p15A ori, *sacB* | Laboratory collection |
| pJQ200SK::*nagA*UD | Plasmid for the deletion of *nagA*, Gm^r^ | This study |
| pJQ200SK:: *dasR*UD | Plasmid for the deletion of *dasR*, Gm^r^ | This study |
| pJQ200SK:: *pheA*UD | Plasmid for the deletion of *pheA*, Gm^r^ | This study |
| pJQ200SK:: *nagE2*UD | Plasmid for the deletion of *nagE2*, Gm^r^ | This study |

*^a^*Km^r^, kanamycin resistant; Gm^r^, gentamicin resistant

**Table S2.** Primers used in this study

| Primer | Sequence (5’-3’) |
| --- | --- |
| *nagA*-UF | CCGCTCGAGAGCAGCAGGGTCAGCGAG (*Xho*I) |
| *nagA*-UR | CCCAAGCTTCTGGACGACGCCCTGTTC (*Hin*dIII) |
| *nagA*-DF | CCCAAGCTTGTCCTCGACGATCACGCTGA (*Hin*dIII) |
| *nagA*-DR | GGACTAGTAACGCCGCACCTTCCTTGA (*Spe*I) |
| *nagA*-VFI | ATCGCATCGGTGACCAACA |
| *nagA*-VRI | GCATTCTCGGCATCCACCT |
| *dasR*-UF | CGGGGCCCCGTTGGCCGCCTTGTAGAA (*Apa*I) |
| *dasR*-UR | CCCAAGCTTTCGAGTTCACCCGATCCTTC (*Hin*dIII) |
| *dasR*-DF | CCCAAGCTTGCGACAGATCCAGCAGCTT (*Hin*dIII) |
| *dasR*-DR | GGACTAGTCCGTCTCTTTGCCGAGGTAA (*Spe*I) |
| *dasR*-VFI | ACCGTCGTCAAGGAAGGT |
| *dasR*-VRI | CGCATCGTCAAATCGTTCTC |
| *pheA*-UF | CGGGGCCCACGGTGGTAAGACGAACTACG (*Apa*I) |
| *pheA*-UR | CCCAAGCTTCGGCTTGTTCTTGCTGGATT (*Hin*dIII) |
| *pheA*-DF | CCCAAGCTTACCTCGCTGCTGATCTTCA (*Hin*dIII) |
| *pheA*-DR | GGACTAGTGCTGATGCGGATACAGTCG (*Spe*I) |
| *pheA*-VFI | CCACATCCAGCAGCTCATC |
| *pheA*-VRI | GCTTGGAGGTCAGGAACATG |
| *nagE2*-UF | CCGCTCGAGGCAGGAAGTTGGCGAAGAAG (*Xho*I) |
| *nagE2*-UR | CGGGATCCGCTCGGCGATCATGGTCAT (*Bam*HI) |
| *nagE2*-DF | CGGGATCCTGGTGAACTCGCCGAACA (*Bam*HI) |
| *nagE2*-DR | GGACTAGTAGGGCAGGTTGGAATTGACT (*Spe*I) |
| *nagE2*-VFI | CGACGAACAACACCGAGAC |
| *nagE2*-VRI | TCAATCCCTACATCAGCATCCT |
| *lecA*-real-F | AACTGGCGTTGGTGTCGA |
| *lecA*-real-R | GTCTGGCTGGAGGAGAACA |
| *lecC*-real-F | CTGATCGTCTGCGGCATCTA |
| *lecC*-real-R | CGCCATCCGATGTTCTTCTG |
| 3655-16S-real-F | GCGGTAATACGAAGGGTGC |
| 3655-16S-real-R | TTCCGCTACCCTCTACCG |


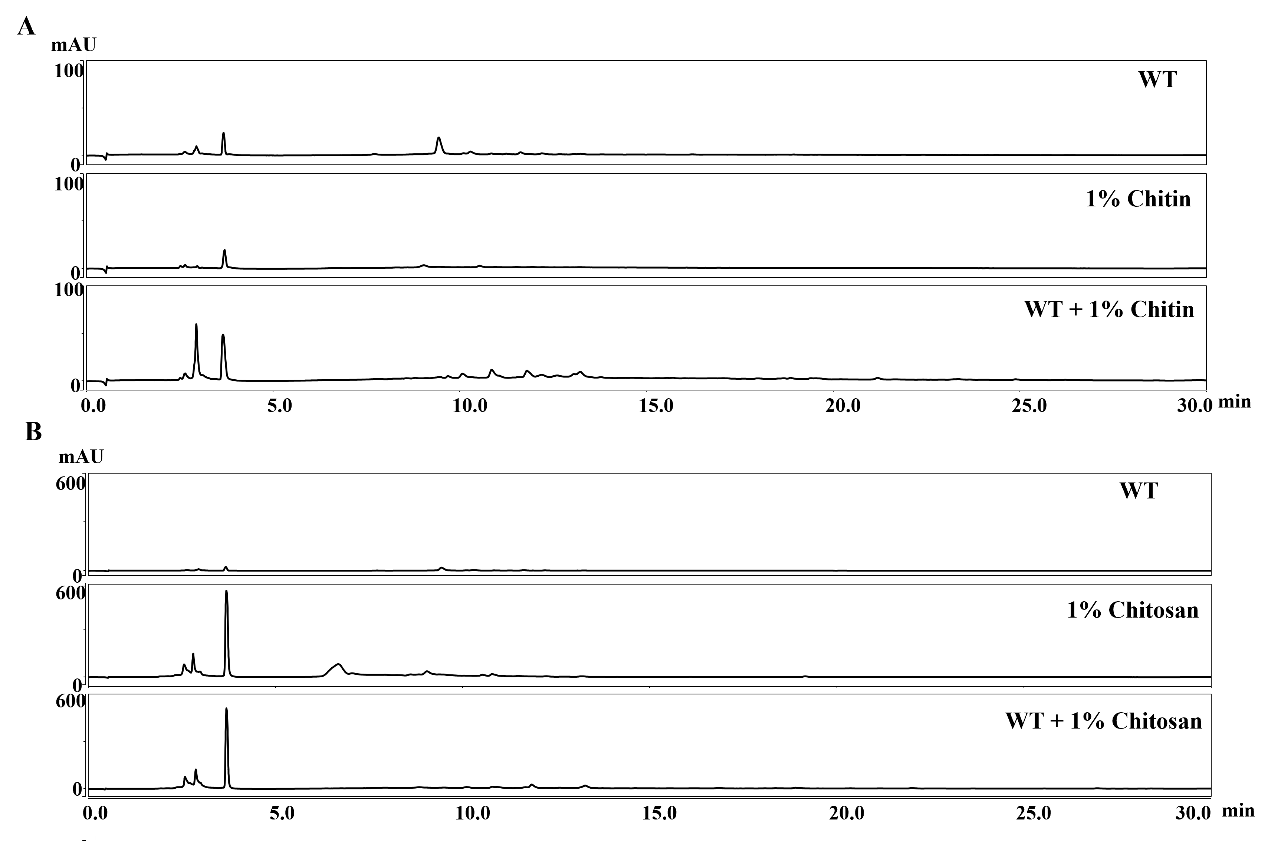


**Figure S1** HPLC analysis of the culture extracts from *Lysobacter* sp. 3655 wild type (WT) grown in M813m medium with Chitin (A) or Chitosan (B). The M813m medium with Chitin (1% Chitin) or Chitosan (1% Chitosan) was used as the blank control.


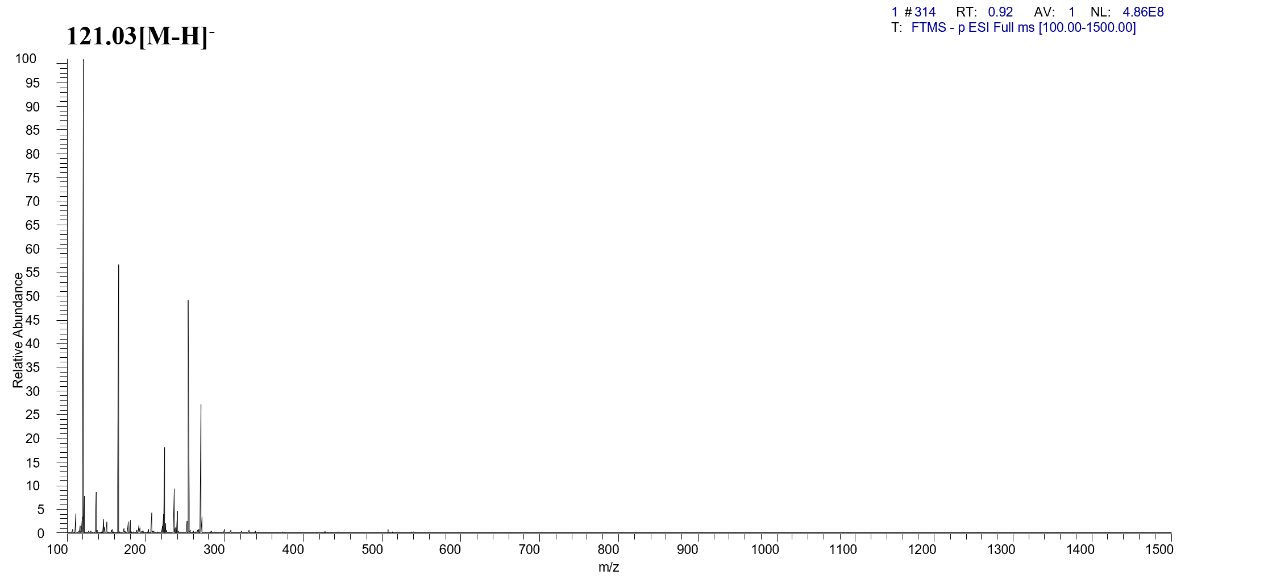


**Figure S2** Mass spectrometry analysis of Compound **1**

**
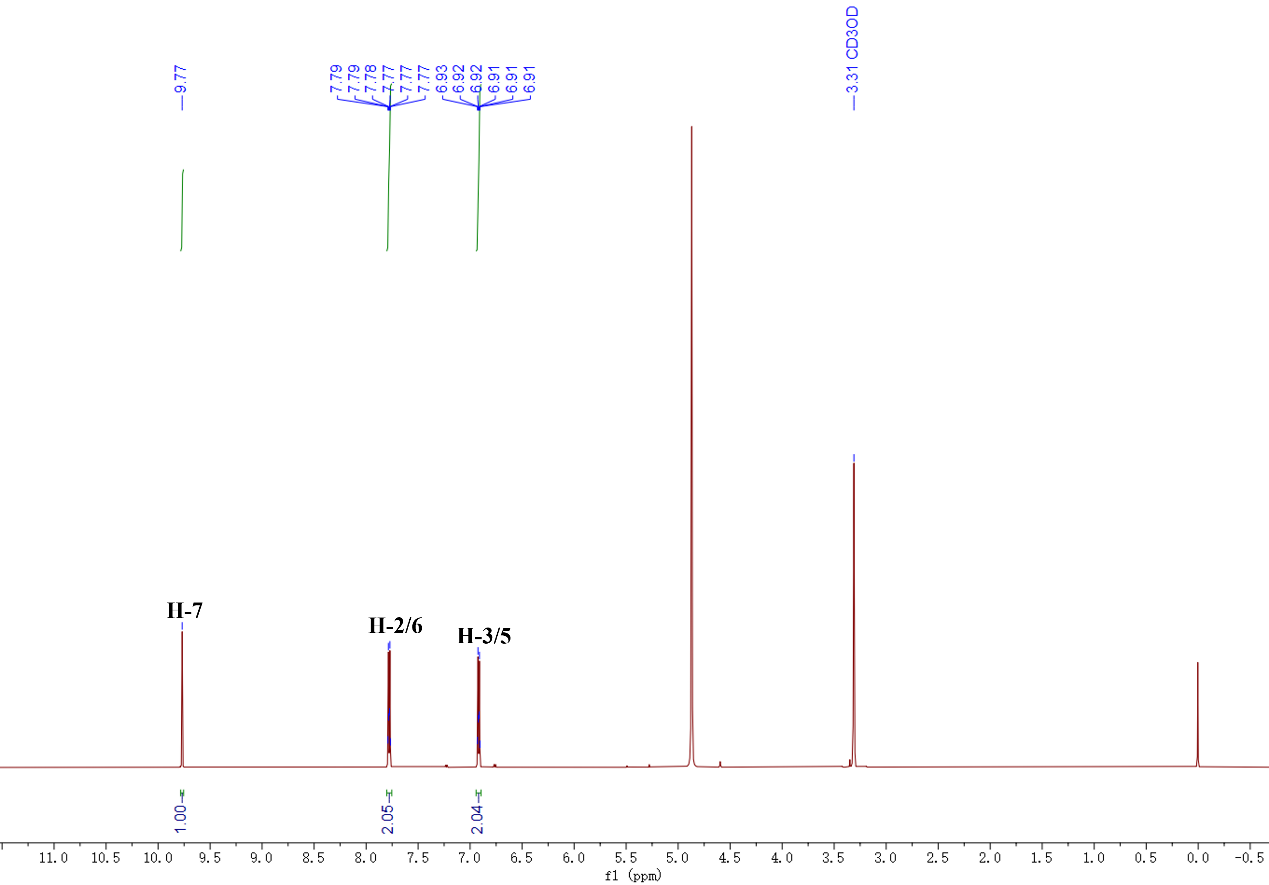
**

**Figure S3** ^1^H-NMR spectra of Compound **1**


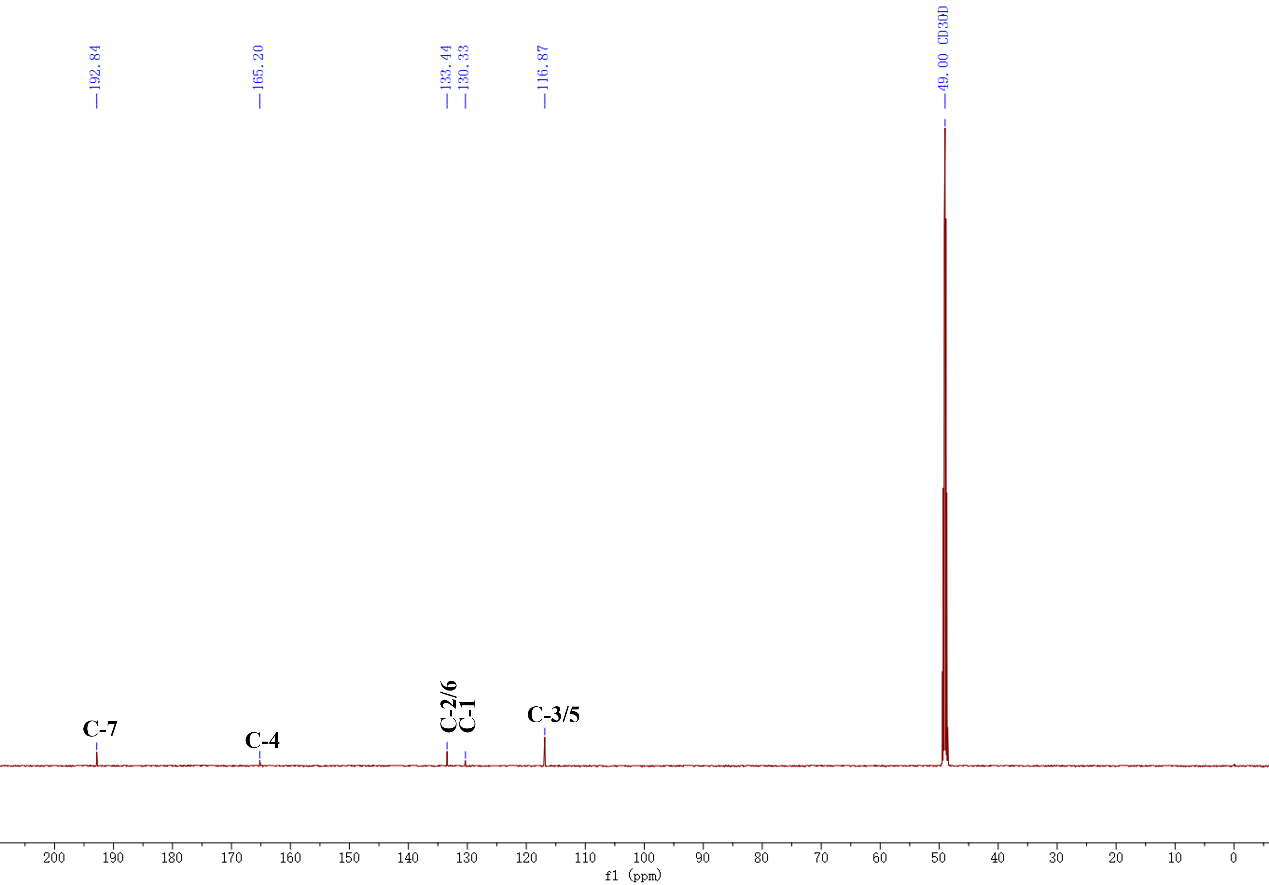


**Figure S4** ^13^C-NMR spectra of Compound **1**


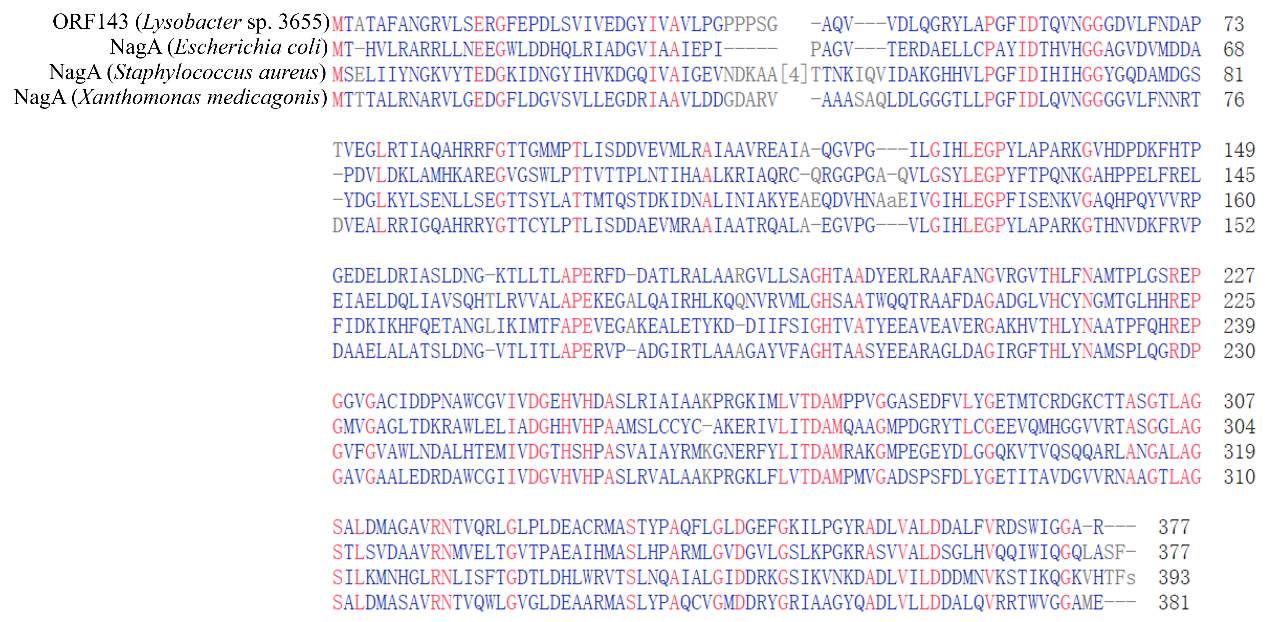


**Figure S5** Alignment of the amino acid sequence of ORF143 in *Lysobacter* sp. 3655 with *N*-acetylglucosamine-6-phosphate deacetylase (NagA) from *Escherichia coli*, *Staphylococcus aureus* and *Xanthomonas medicagonis*. The red color indicates identical columns and blue indicates high conserved ones.

**
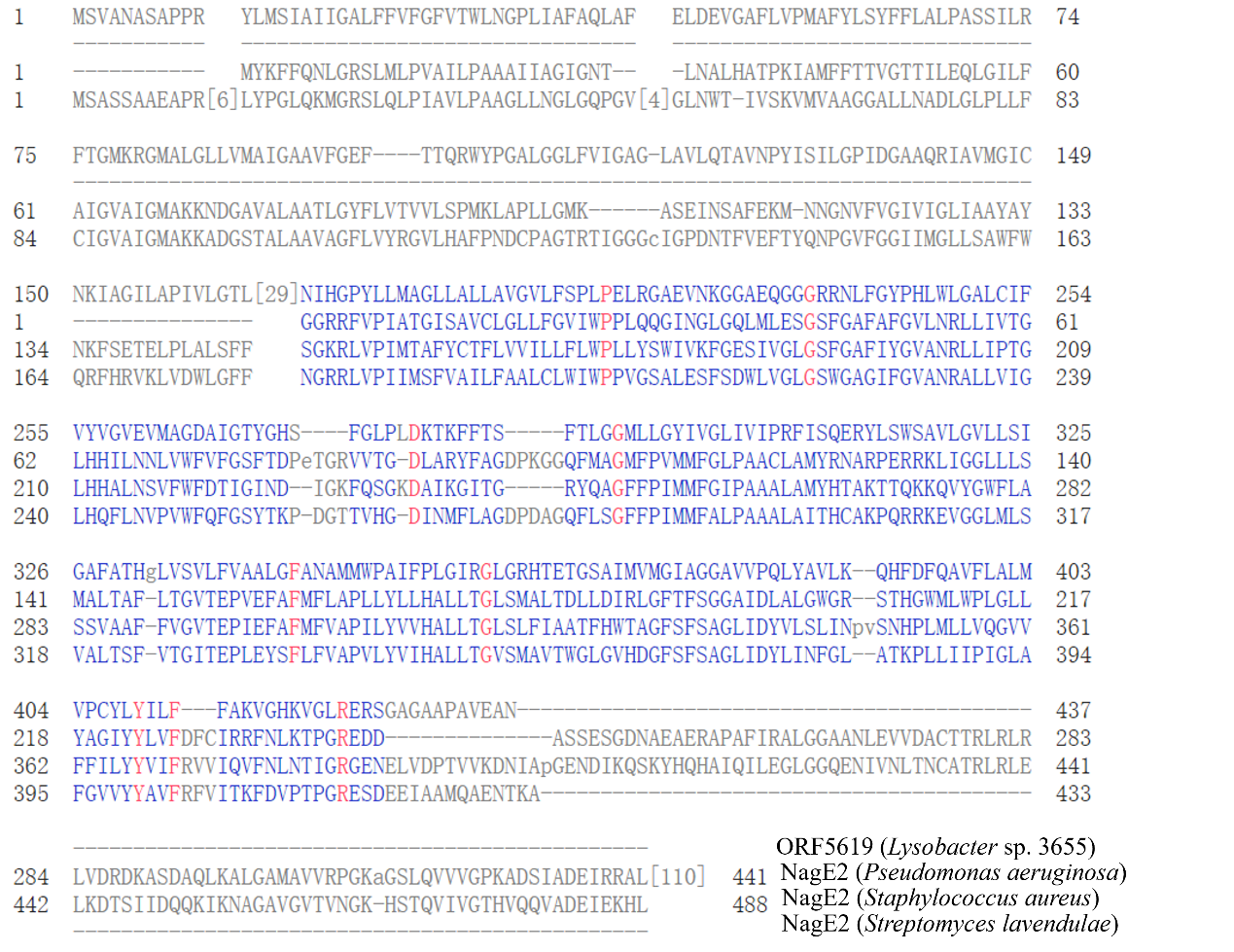
**

**Figure S6** Alignment of the amino acid sequence of ORF5619 in *Lysobacter* sp. 3655 with GlcNAc permease (NagE2) from *Pseudomonas aeruginosa*, *Staphylococcus aureus* and *Streptomyces lavendulae*. The red color indicates identical columns and blue indicates high conserved ones.


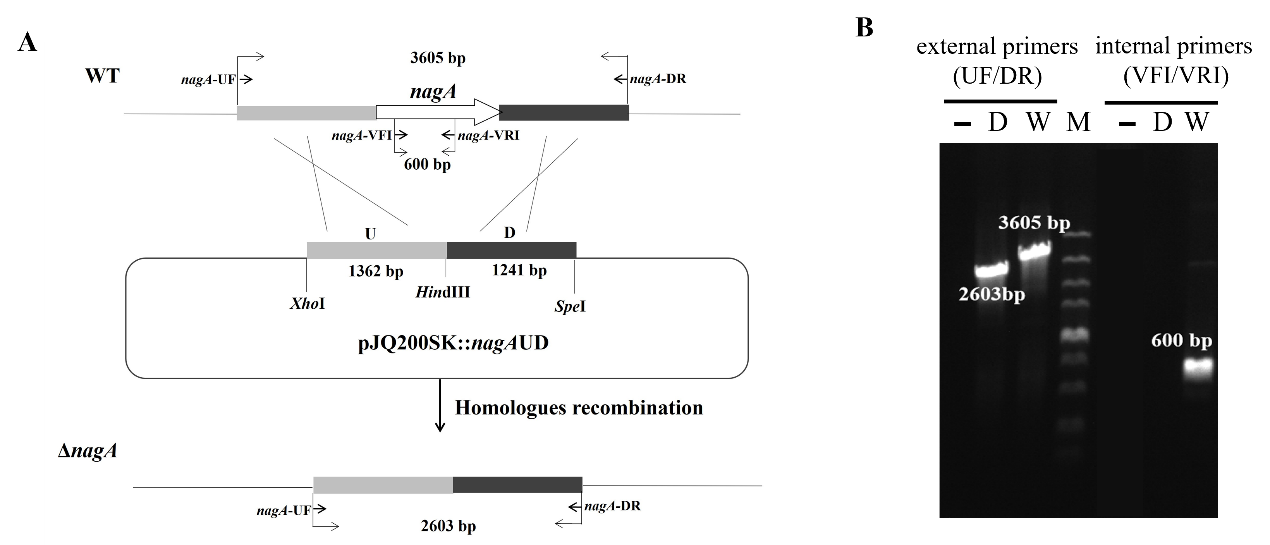


**Figure S7** Confirmation of the deletion mutant of *nagA* (*orf143*). (A) Schematic representation of the deletion mutant in *Lysobacter* sp. 3655 (WT). (B) PCR verification of the strain Δ*nagA*. -: negative control, H_2_O was used as the template. D: the gDNA of the deletion mutant was used as the template by external primers *nagA*-UF/*nagA*-DR (a DNA fragment of 2603 bp was amplified) and internal primers *nagA*-VFI/*nagA*-VRI (no DNA fragment was amplified). W: positive control, the gDNA of WT was used as the template by external primers *nagA*-UF/*nagA*-DR (a DNA fragment of 3605 bp was amplified) and internal primers *nagA*-VFI/*nagA*-VRI (a DNA fragment of 600 bp was amplified). M: DNA marker. U, the upstream region of *nagA*; D, the downstream region of *nagA*.


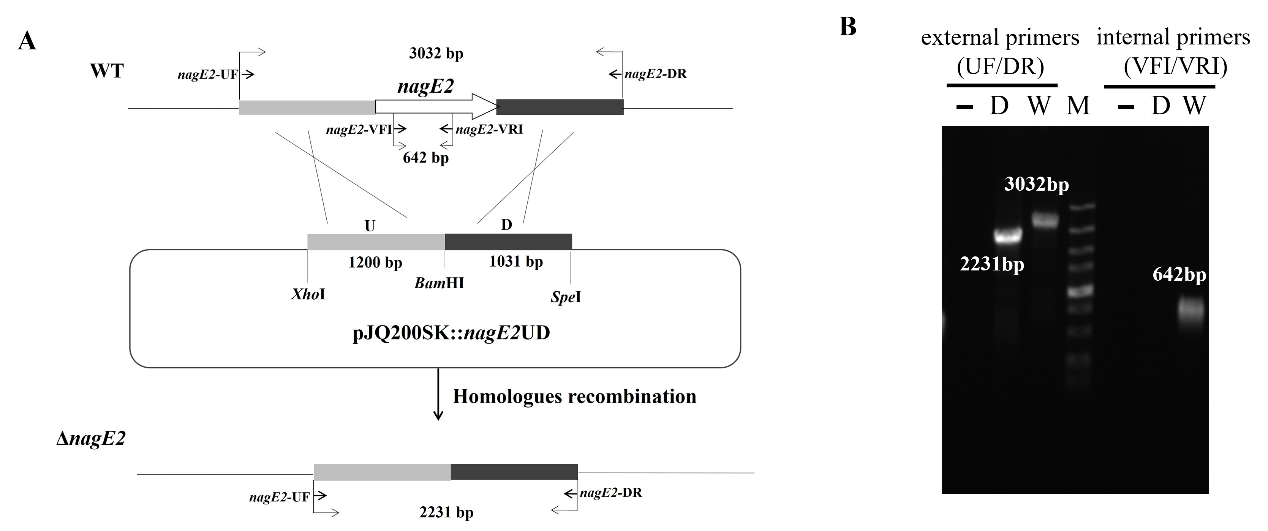


**Figure S8** Confirmation of the deletion mutant of *nagE2* (*orf5619*). (A) Schematic representation of the deletion mutant in *Lysobacter* sp. 3655 (WT). (B) PCR verification of the strain Δ*nagE2*. -: negative control, H_2_O was used as the template. D: the gDNA of the deletion mutant was used as the template by external primers *nagE2*-UF/*nagE2*-DR (a DNA fragment of 2231 bp was amplified) and internal primers *nagE2*-VFI/*nagE2*-VRI (no DNA fragment was amplified). W: positive control, the gDNA of WT was used as the template by external primers *nagE2*-UF/*nagE2*-DR (a DNA fragment of 3032 bp was amplified) and internal primers *nagE2*-VFI/*nagE2*-VRI (a DNA fragment of 642 bp was amplified). M: DNA marker. U, the upstream region of *nagE2*; D, the downstream region of *nagE2*.


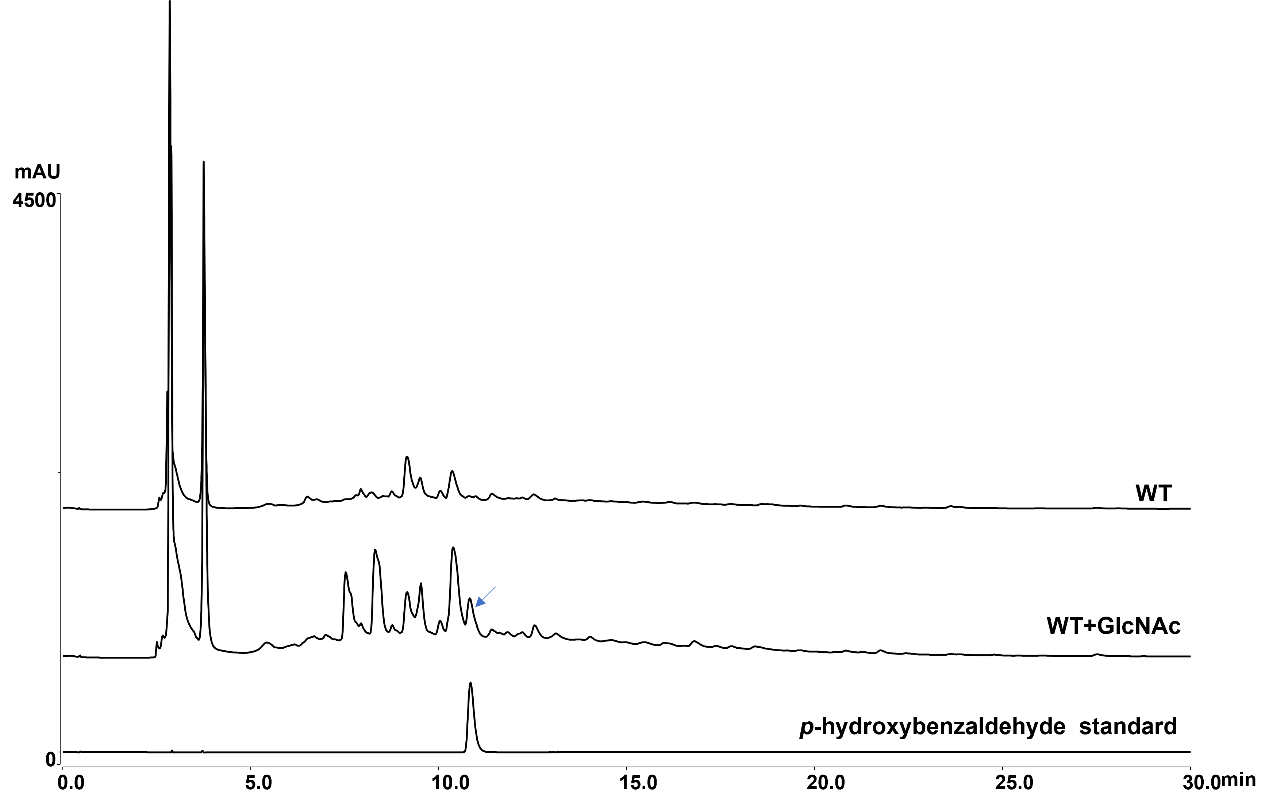


**Figure S9** HPLC analysis of the culture extracts from *Lysobacter* sp. 3655 wild type (WT) grown in YME medium without or with GlcNAc. Pure *p*-hydroxybenzaldehyde was used as standard. The arrow indicated the signal peak of *p*-hydroxybenzaldehyde.


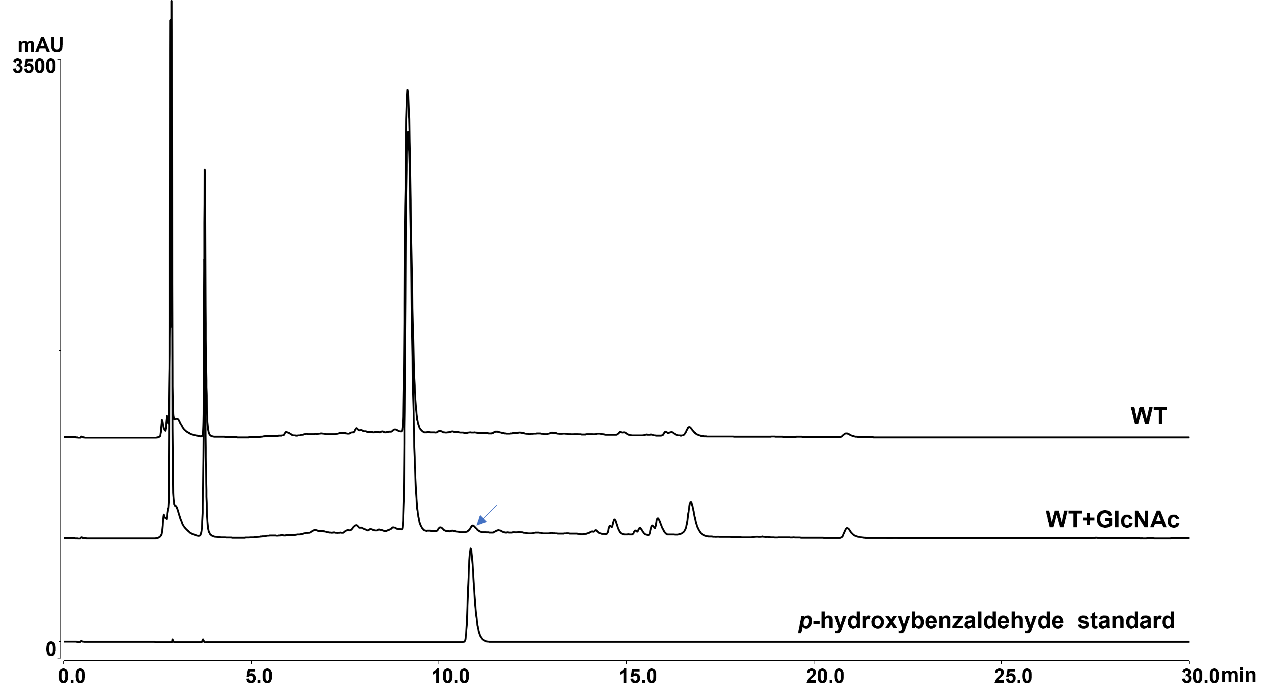


**Figure S10** HPLC analysis of the culture extracts from *Lysobacter* sp. 3655 wild type (WT) grown in 1/10 TSB medium without or with GlcNAc. Pure *p*-hydroxybenzaldehyde was used as standard. The arrow indicated the signal peak of *p*-hydroxybenzaldehyde.


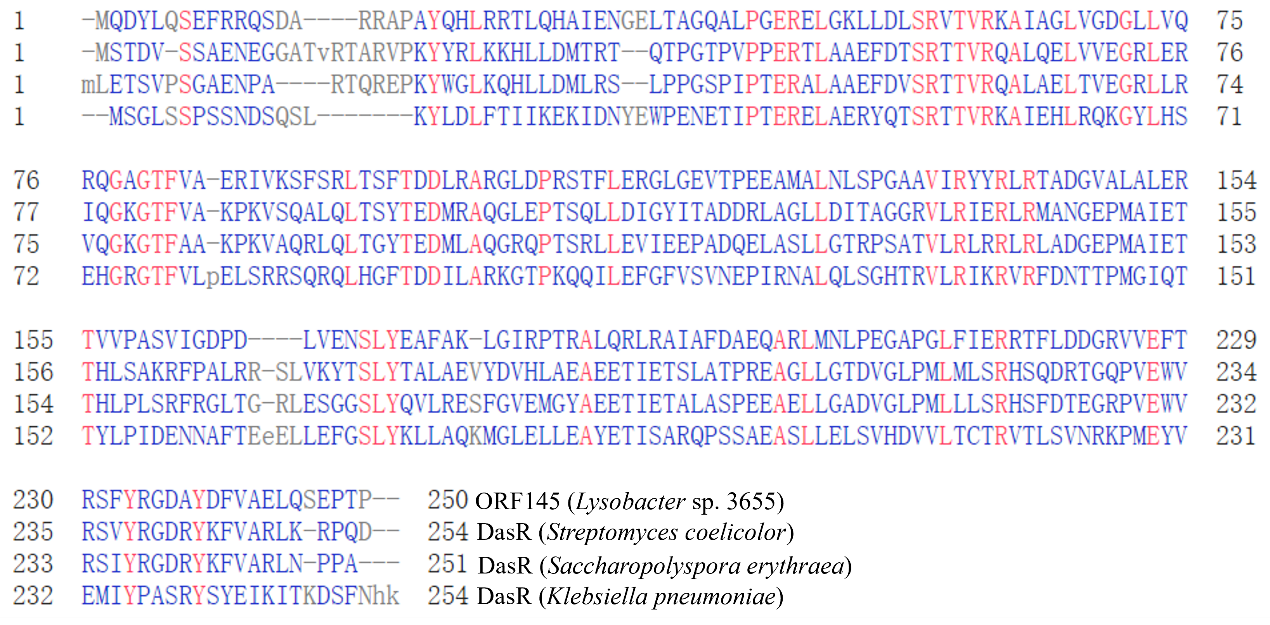


**Figure S11** Alignment of the amino acid sequence of ORF145 in *Lysobacter* sp. 3655 with DasR regulator from *Streptomyces coelicolor*, *Saccharopolyspora erythraea* and *Klebsiella pneumoniae*. The red color indicates identical columns and blue indicates high conserved ones.


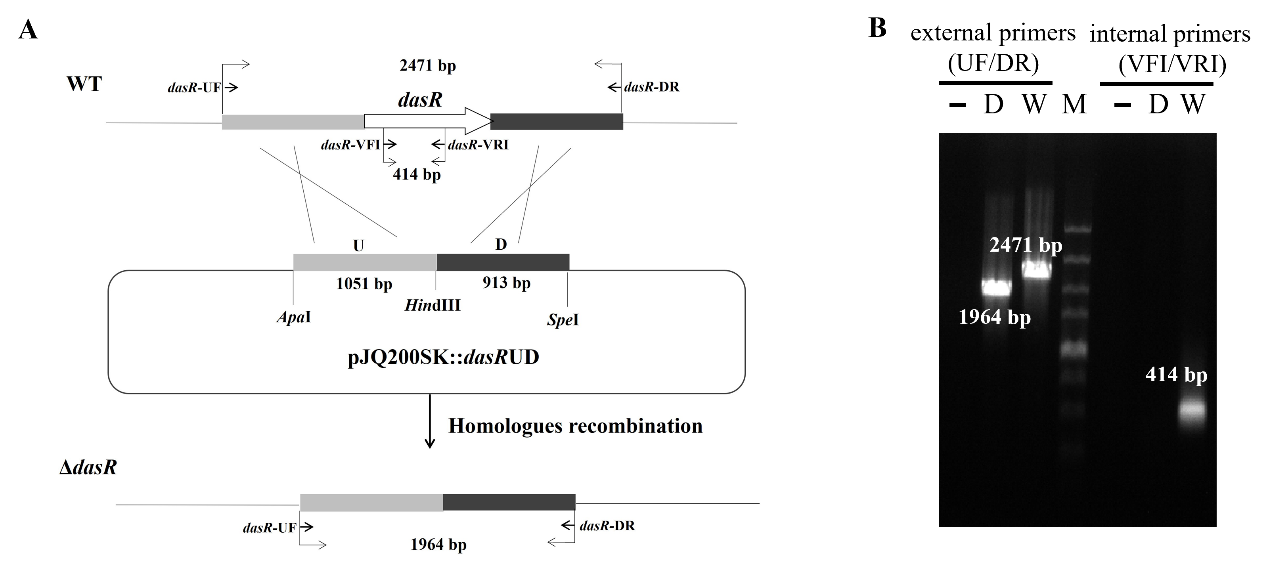


**Figure S12** Confirmation of the deletion mutant of *dasR* (*orf145*). (A) Schematic representation of the deletion mutant in *Lysobacter* sp. 3655 (WT). (B) PCR verification of the strain Δ*dasR*. -: negative control, H_2_O was used as the template. D: the gDNA of the deletion mutant was used as the template by external primers *dasR*-UF/*dasR*-DR (a DNA fragment of 1964 bp was amplified) and internal primers *dasR*-VFI/*dasR*-VRI (no DNA fragment was amplified). W: positive control, the gDNA of WT was used as the template by external primers *dasR*-UF/*dasR*-DR (a DNA fragment of 2471 bp was amplified) and internal primers *dasR*-VFI/*dasR*-VRI (a DNA fragment of 414 bp was amplified). M: DNA marker. U, the upstream region of *dasR*; D, the downstream region of *dasR*.


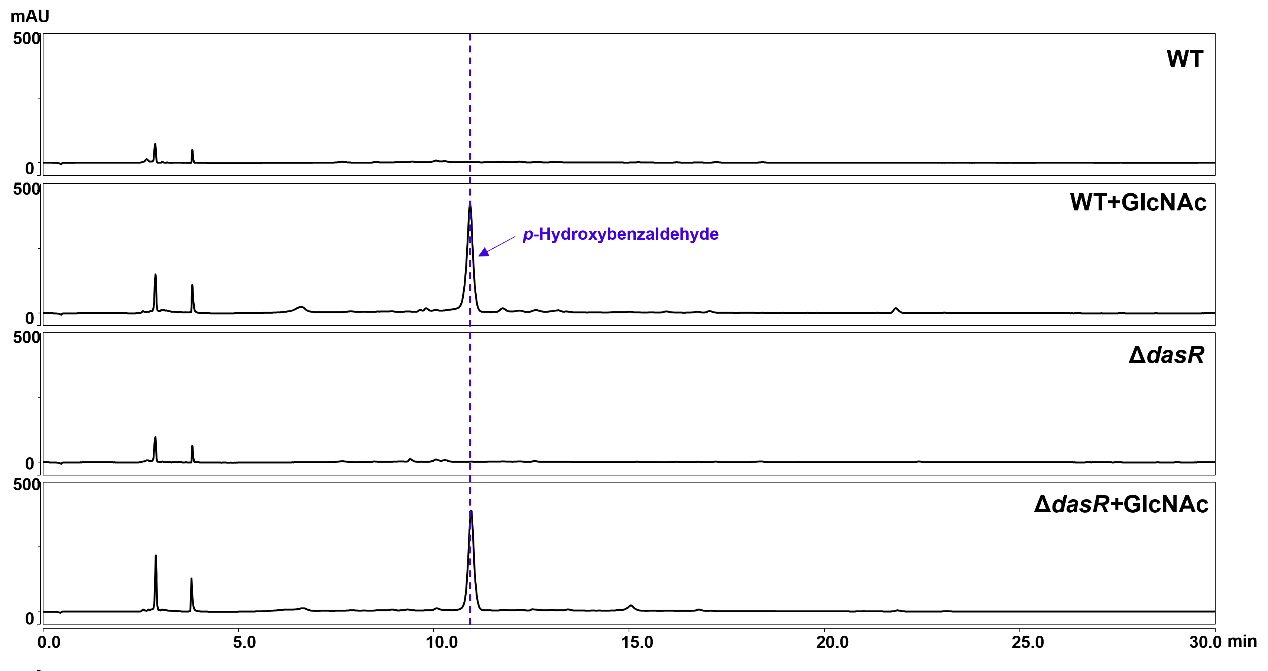


**Figure S13** HPLC analysis of *p*-hydroxybenzaldehyde production in WT, WT with GlcNAc (WT+GlcNAc), Δ*dasR* and Δ*dasR* with GlcNAc (Δ*dasR*+GlcNAc) grown in M813m medium for 72 h.


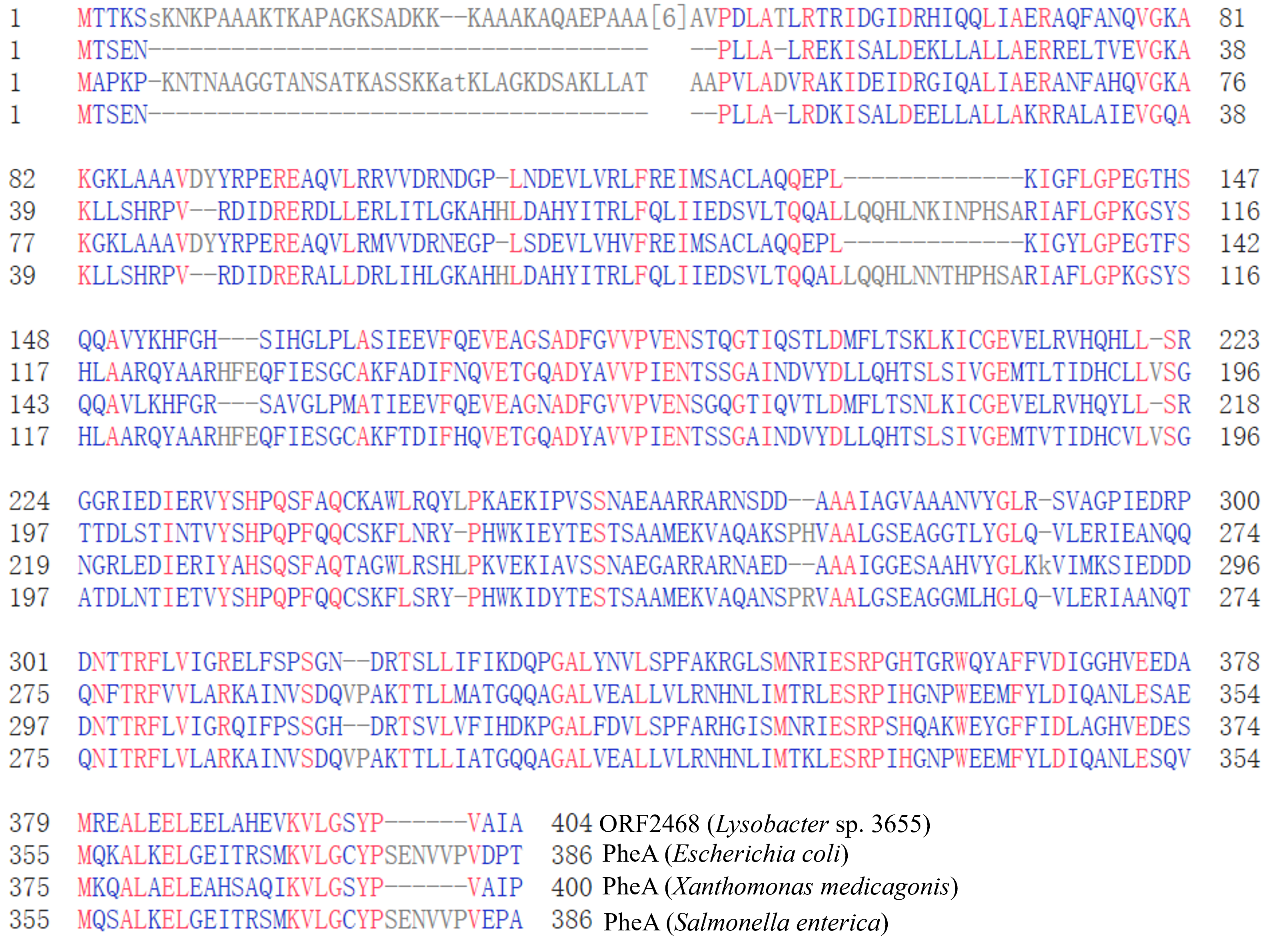


**Figure S14** Alignment of the amino acid sequence of ORF2468 in strain 3655 with bifunctional chorismate mutase/prephenate dehydratase (PheA) from *Escherichia coli*, *Xanthomonas medicagonis* and *Salmonella enterica*. The red color indicates identical columns and blue indicates high conserved ones.


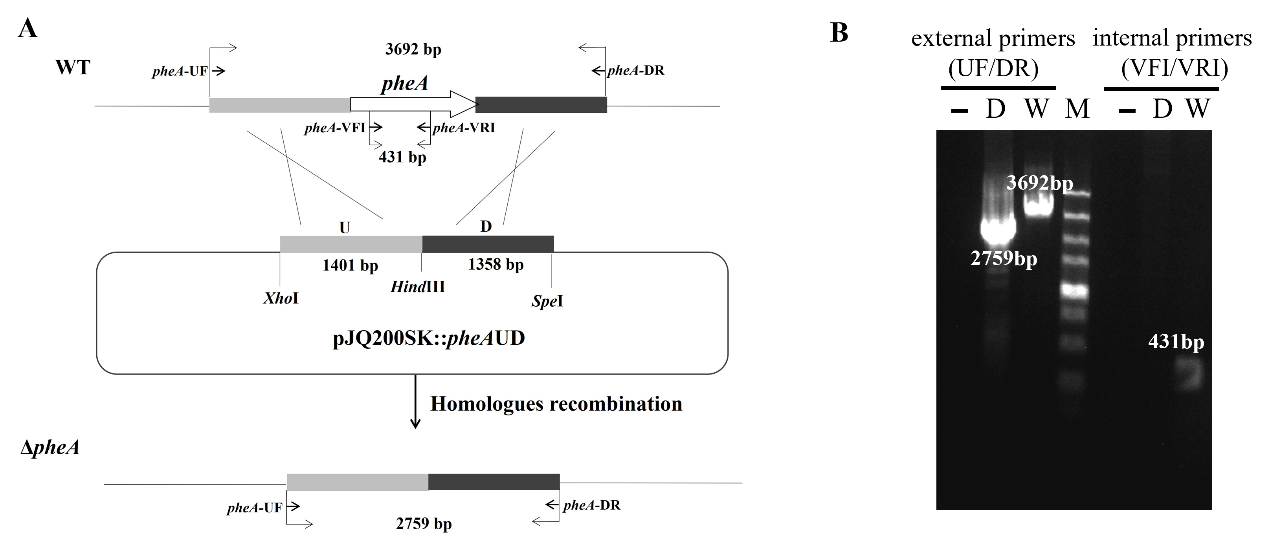


**Figure S15** Confirmation of the deletion mutant of *pheA* (*orf2468*). (A) Schematic representation of the deletion mutant in *Lysobacter* sp. 3655 (WT). (B) PCR verification of the strain Δ*pheA*. -: negative control, H_2_O was used as the template. D: the gDNA of the deletion mutant was used as the template by external primers *pheA*-UF/ *pheA*-DR (a DNA fragment of 2759 bp was amplified) and internal primers *dasR*-VFI/*dasR*-VRI (no DNA fragment was amplified). W: positive control, the gDNA of WT was used as the template by external primers *pheA*-UF/ *pheA*-DR (a DNA fragment of 3692 bp was amplified) and internal primers *pheA*-VFI/ *pheA*-VRI (a DNA fragment of 431 bp was amplified). M: DNA marker. U, the upstream region of *pheA*; D, the downstream region of *pheA*.


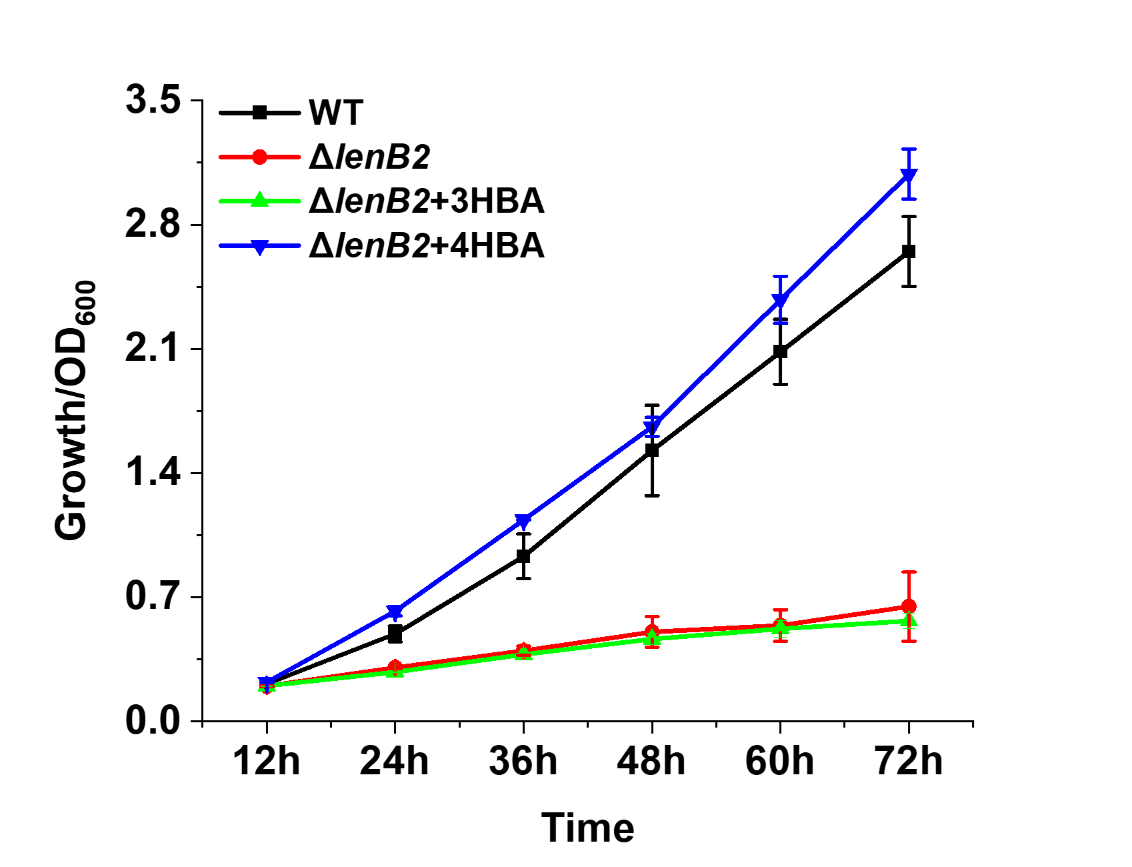


**Figure S16** The growth curve of WT (black line), Δ*lenB2* (red line), Δ*lenB2* with 3-HBA (green line) and Δ*lenB2* with 4-HBA (blue line) grown in M813m-Fe medium.


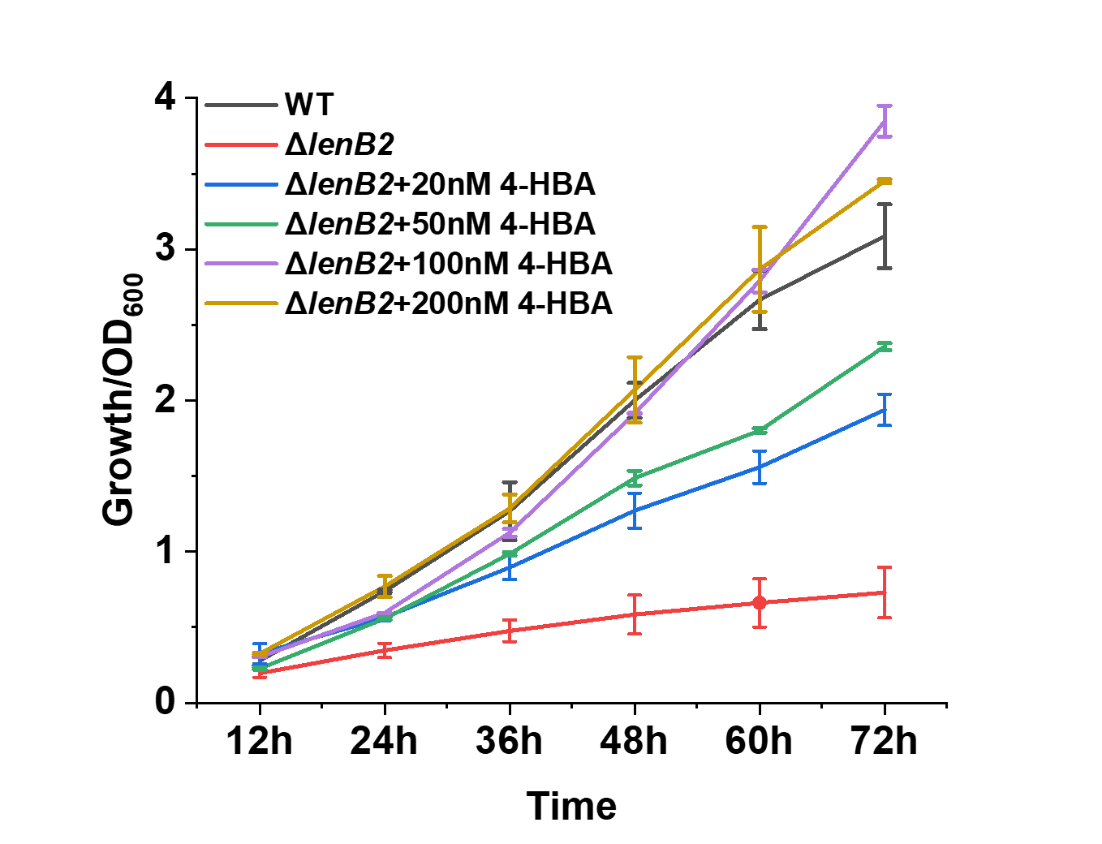


**Figure S17** The growth curve of WT (black line), Δ*lenB2* (red line), Δ*lenB2* with 20 nM 4-HBA (blue line), Δ*lenB2* with 50 nM 4-HBA (green line), Δ*lenB2* with 100 nM 4-HBA (purple line) and Δ*lenB2* with 200 nM 4-HBA (brown line) grown in M813m-Fe medium.


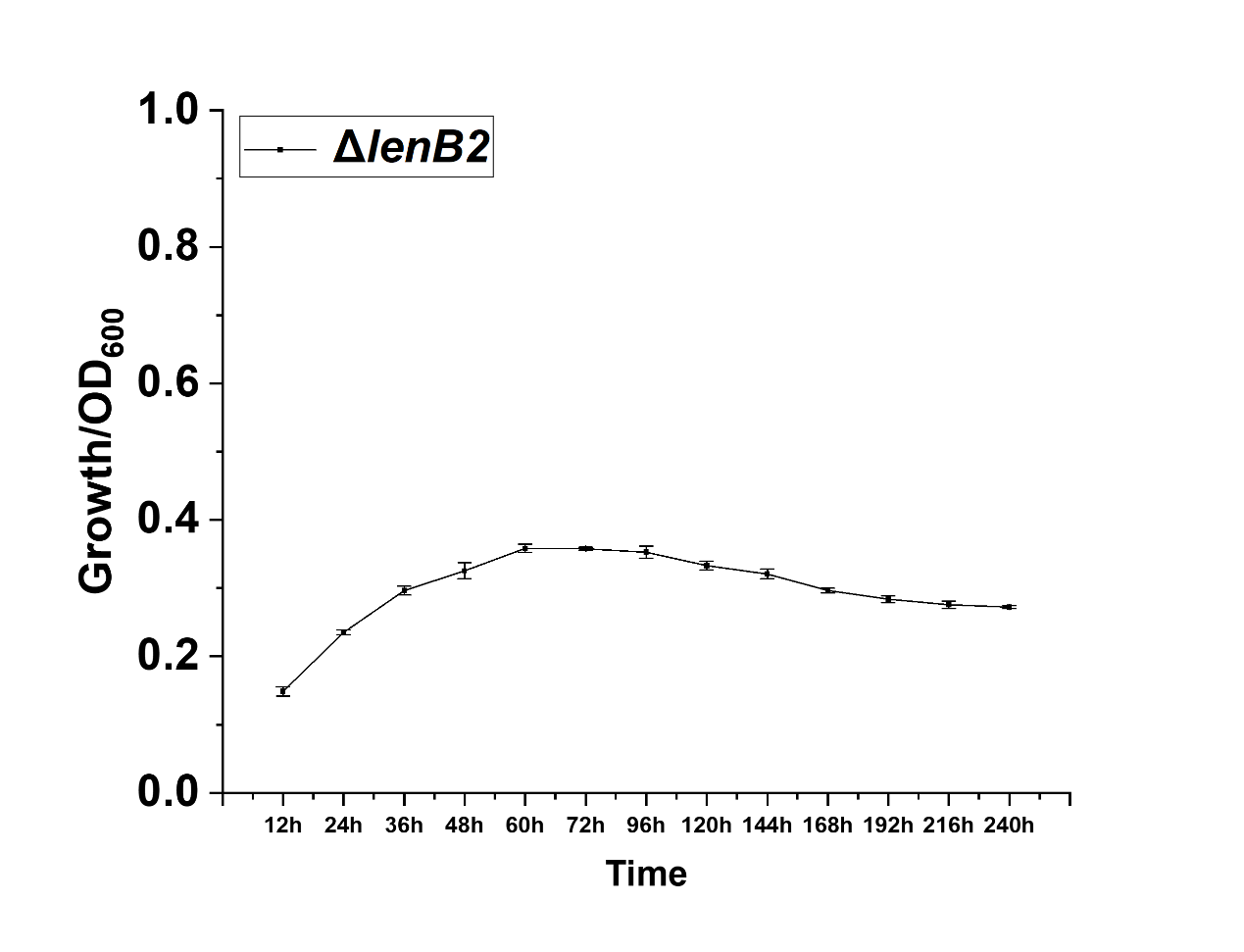


**Figure S18** The growth curve of Δ*lenB2* cultured in M813m-Fe medium.


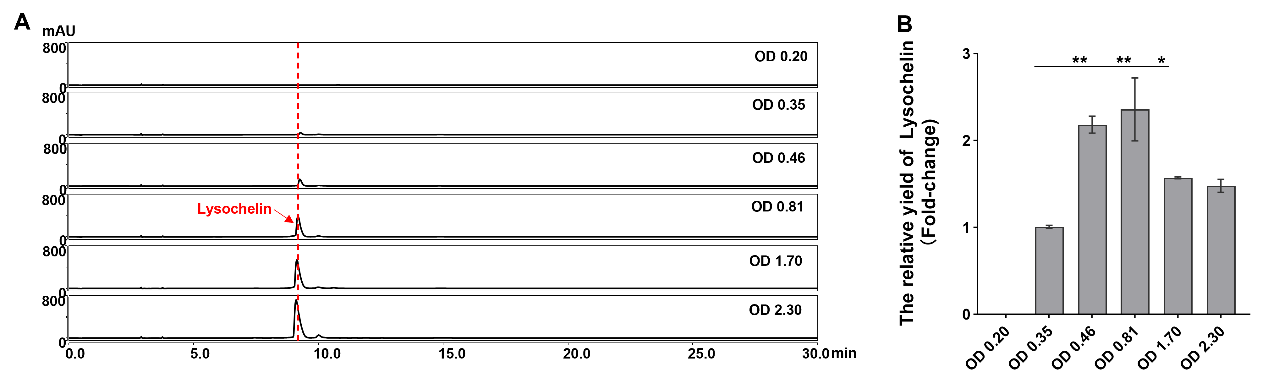


**Figure S19** The effect of cell density on lysochelin production. (A) HPLC analysis of lysochelin production in *Lysobacter* sp. 3655 grown in M813m-Fe medium at the OD_600_ value of 0.20, 0.35, 0.46, 0.81, 1.70 and 2.30. (B) Quantification of lysochelin in the cultures from (A). Data are presented as averages of three independent experiments, each conducted in triplicate. Statistical analyses were conducted using one-way analysis of variance (ANOVA) followed by Tukey’s post-hoc test. **p* < 0.05; ***p* < 0.01


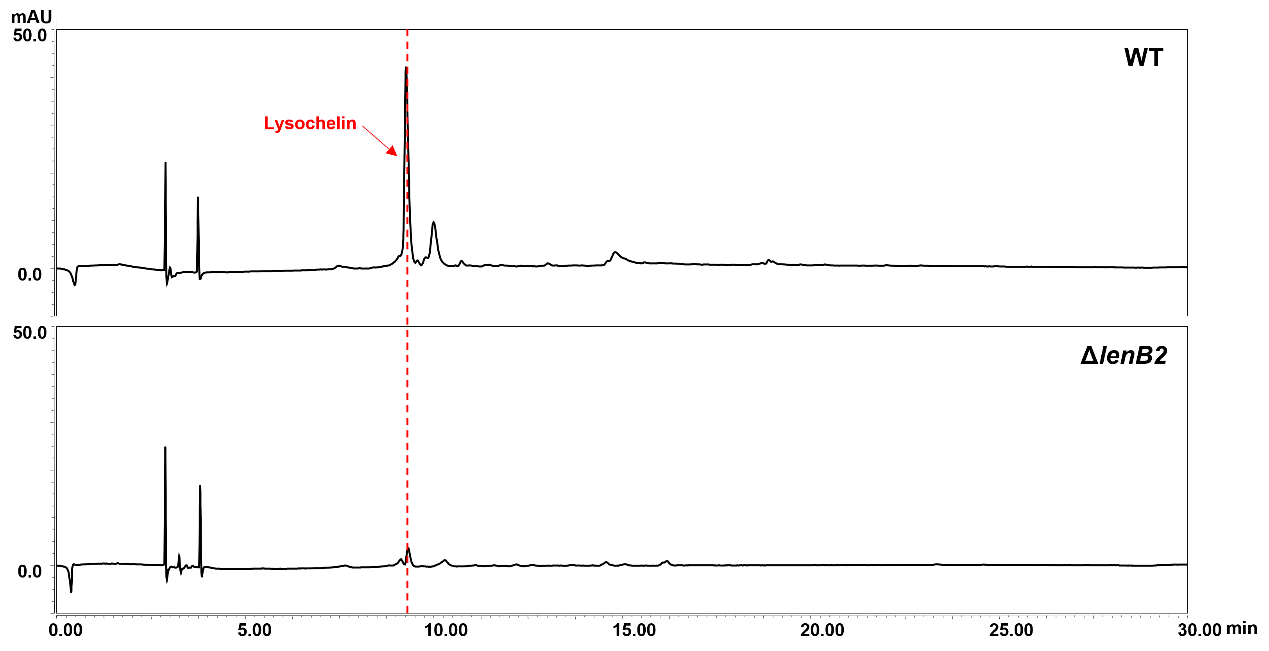


**Figure S20** HPLC analysis of lysochelin production in WT and Δ*lenB2* grown in M813m-Fe medium at the OD_600_ value of 0.35.


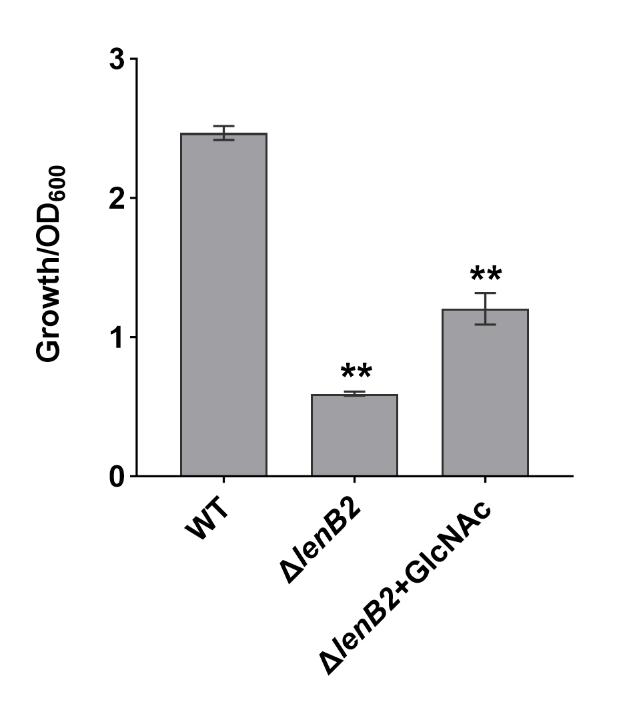


**Figure S21** The OD_600_ value of WT, Δ*lenB2* and Δ*lenB2* with GlcNAc (Δ*lenB2*+GlcNAc) grown in M813m-Fe medium for 72 h. WT was used as the control strain. Data are presented as averages of three independent experiments, each conducted in triplicate. Statistical analyses were conducted using one-way analysis of variance (ANOVA) followed by Tukey’s post-hoc test. ***p* < 0.01


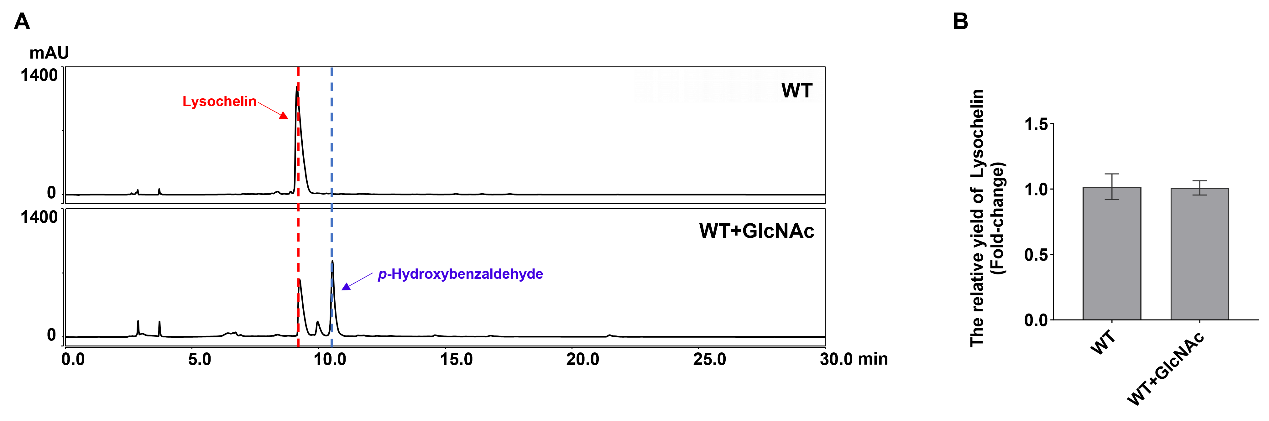


**Figure S22** Effect of GlcNAc on lysochelin production. (A) HPLC analysis of lysochelin production in WT and WT with GlcNAc grown in M813m-Fe medium for 72 h. (B) Quantification of lysochelin in the cultures from (A). Data are presented as averages of three independent experiments, each conducted in triplicate. Statistical analyses were conducted using one-way analysis of variance (ANOVA) followed by Tukey’s post-hoc test.


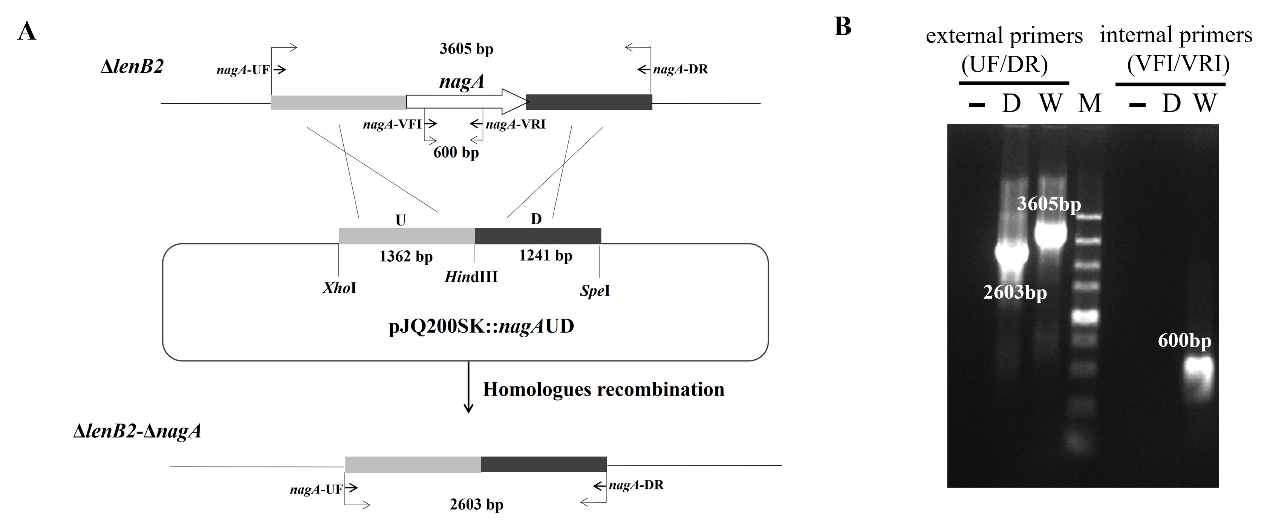


**Figure S23** Confirmation of the deletion mutant of *nagA* (*orf143*) in Δ*lenB2*. (A) Schematic representation of the deletion mutant in Δ*lenB2*. (B) PCR verification of the strain Δ*lenB2*-Δ*nagA*. -: negative control, H_2_O was used as the template. D: the gDNA of Δ*lenB2*-Δ*nagA* was used as the template by external primers *nagA*-UF/*nagA*-DR (a DNA fragment of 2603 bp was amplified) and internal primers *nagA*-VFI/*nagA*-VRI (no DNA fragment was amplified). W: positive control, the gDNA of Δ*lenB2* was used as the template by external primers *nagA*-UF/*nagA*-DR (a DNA fragment of 3605 bp was amplified) and internal primers *nagA*-VFI/*nagA*-VRI (a DNA fragment of 600 bp was amplified). M: DNA marker. U, the upstream region of *nagA*; D, the downstream region of *nagA*.


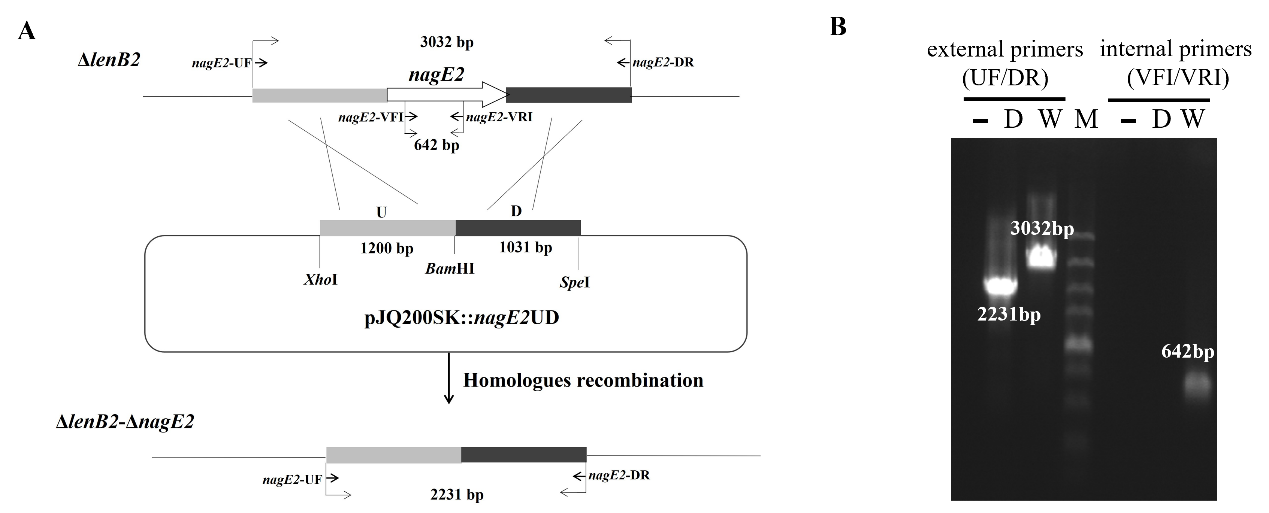


**Figure S24** Confirmation of the deletion mutant of *nagE2* (*orf5619*) in Δ*lenB2*. (A) Schematic representation of the deletion mutant in Δ*lenB2*. (B) PCR verification of the strain Δ*lenB2*-Δ*nagE2*. -: negative control, H_2_O was used as the template. D: the gDNA of Δ*lenB2*-Δ*nagE2* was used as the template by external primers *nagE2*-UF/*nagE2*-DR (a DNA fragment of 2231 bp was amplified) and internal primers *nagE2*-VFI/*nagE2*-VRI (no DNA fragment was amplified). W: positive control, the gDNA of Δ*lenB2* was used as the template by external primers *nagE2*-UF/*nagE2*-DR (a DNA fragment of 3032 bp was amplified) and internal primers *nagE2*-VFI/*nagE2*-VRI (a DNA fragment of 642 bp was amplified). M: DNA marker. U, the upstream region of *nagE2*; D, the downstream region of *nagE2*.


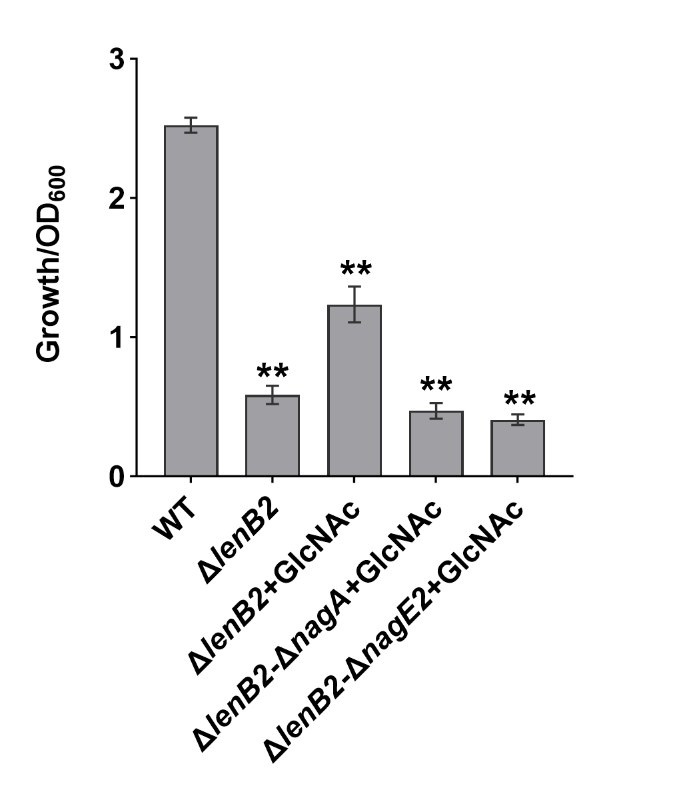


**Figure S25** The OD_600_ value of WT, Δ*lenB2*, Δ*lenB2* with GlcNAc (Δ*lenB2*+GlcNAc), Δ*lenB2*-Δ*nagA* with GlcNAc (Δ*lenB2*-Δ*nagA+*GlcNAc) and Δ*lenB2*-Δ*nagE2* with GlcNAc (Δ*lenB2*-Δ*nagE2*+GlcNAc) grown in M813m-Fe medium for 72 h. WT was used as the control strain. Data are presented as averages of three independent experiments, each conducted in triplicate. Statistical analyses were conducted using one-way analysis of variance (ANOVA) followed by Tukey’s post-hoc test. ***p* < 0.01


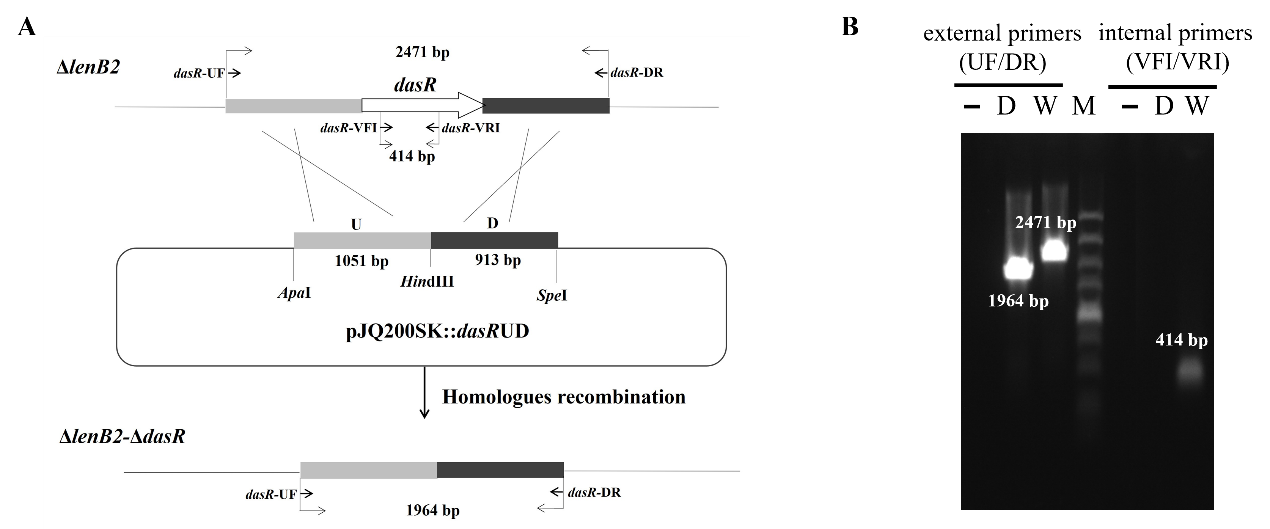


**Figure S26** Confirmation of the deletion mutant of *dasR* (*orf145*) in Δ*lenB2*. (A) Schematic representation of the deletion mutant in Δ*lenB2*. (B) PCR verification of the strain Δ*lenB2*-Δ*dasR*. -: negative control, H_2_O was used as the template. D: the gDNA of Δ*lenB2*-Δ*dasR* was used as the template by external primers *dasR*-UF/*dasR*-DR (a DNA fragment of 1964 bp was amplified) and internal primers *dasR*-VFI/*dasR*-VRI (no DNA fragment was amplified). W: positive control, the gDNA of Δ*lenB2* was used as the template by external primers *dasR*-UF/*dasR*-DR (a DNA fragment of 2471 bp was amplified) and internal primers *dasR*-VFI/*dasR*-VRI (a DNA fragment of 414 bp was amplified). M: DNA marker. U, the upstream region of *dasR*; D, the downstream region of *dasR*.


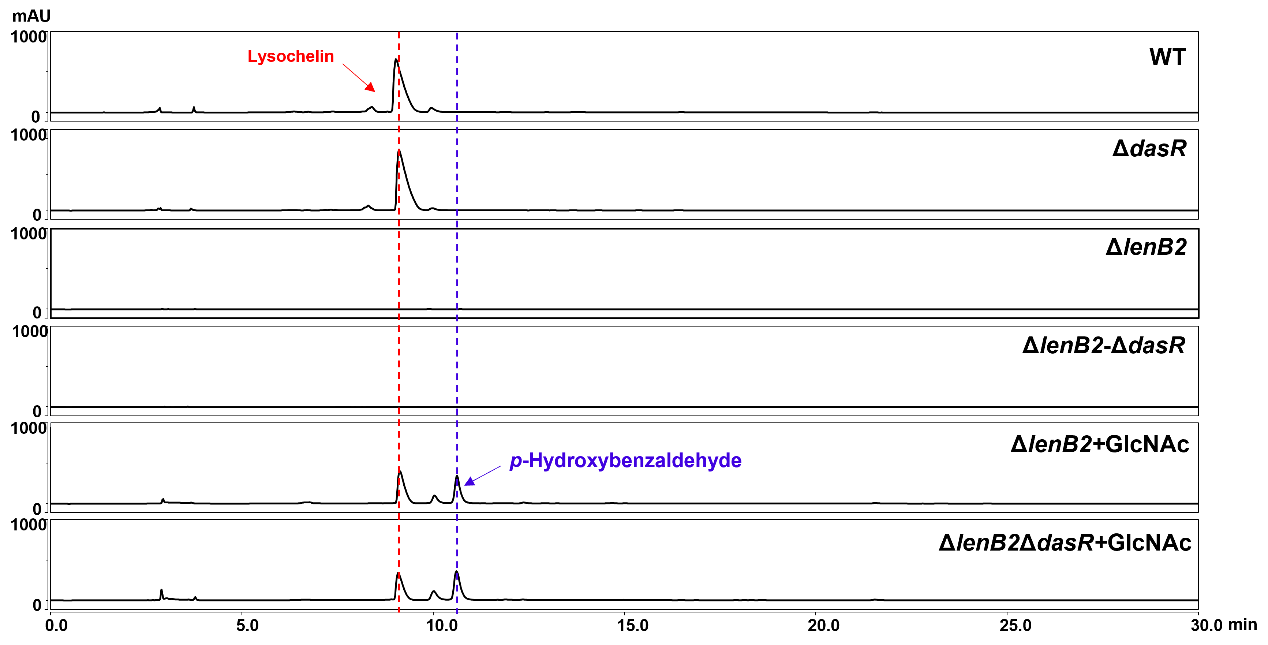


**Figure S27** HPLC analysis of lysochelin and *p*-hydroxybenzaldehyde production in WT, Δ*dasR*, Δ*lenB2*, Δ*lenB2*-Δ*dasR*, Δ*lenB2* with GlcNAc (Δ*lenB2*+GlcNAc) and Δ*lenB2*-Δ*dasR* with GlcNAc (Δ*lenB2*-Δ*dasR+*GlcNAc) grown in M813m-Fe medium for 72 h.


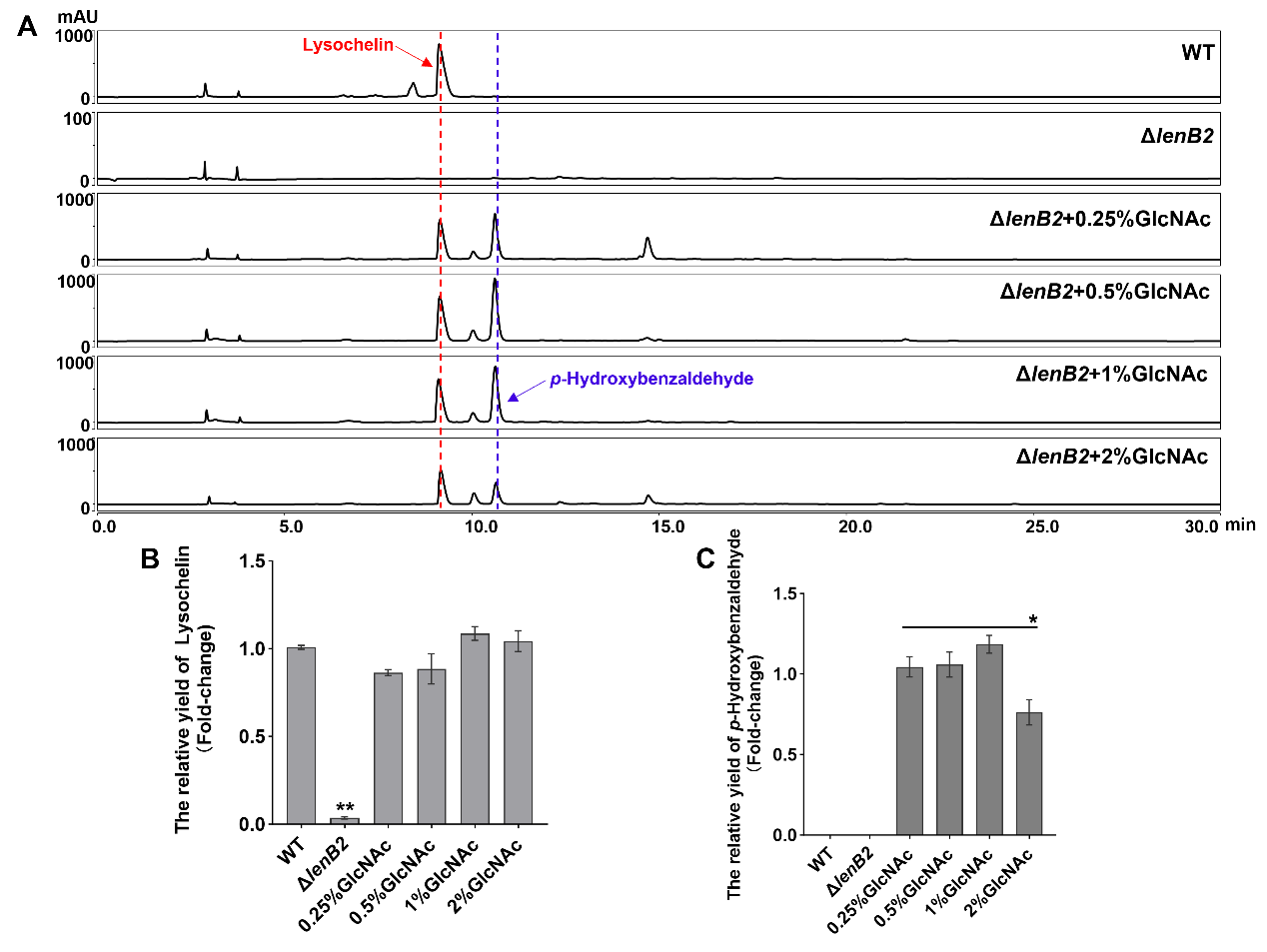


**Figure S28** Effects of the GlcNAc concentration on lysochelin and *p*-hydroxybenzaldehyde production. (A) HPLC analysis of lysochelin and *p*-hydroxybenzaldehyde production in Δ*lenB2* grown in M813m-Fe medium containing different concentrations of GlcNAc (final concentrations of 0, 0.25%, 0.5%, 1% and 2%, w/v) for 72 h. (B) Quantification of lysochelin in the cultures from (A). (C) Quantification of *p*-hydroxybenzaldehyde in the cultures from (A). Data are presented as averages of three independent experiments, each conducted in triplicate. Statistical analyses were conducted using one-way analysis of variance (ANOVA) followed by Tukey’s post-hoc test. **p* < 0.05; ***p* < 0.01


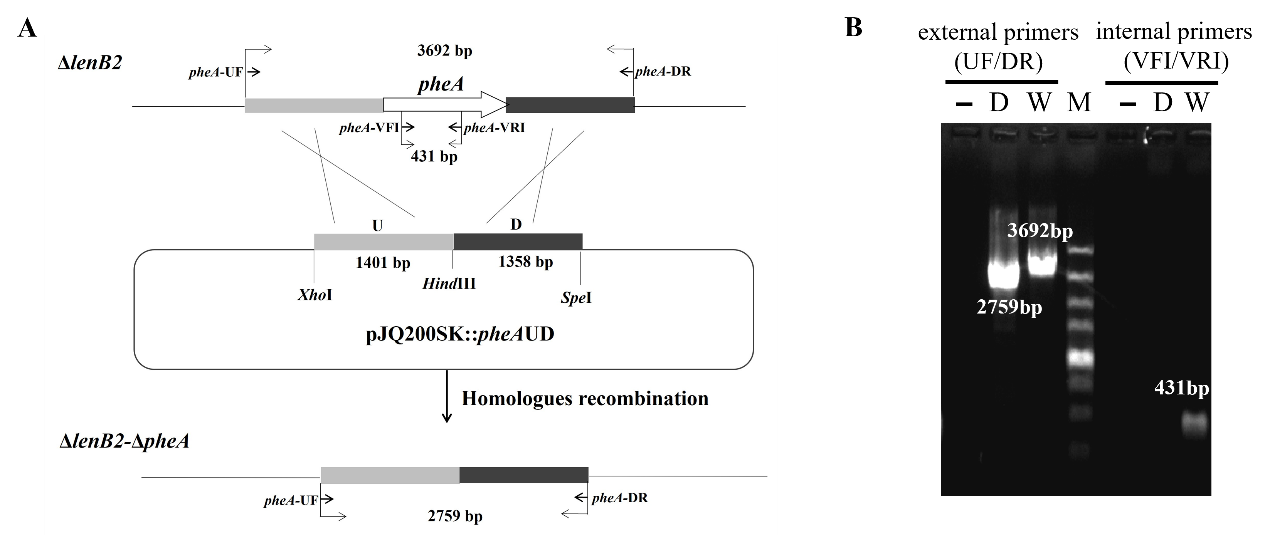


**Figure S29** Confirmation of the deletion mutant of *pheA* (*orf2468*) in Δ*lenB2*. (A) Schematic representation of the deletion mutant in *ΔlenB2*. (B) PCR verification of the strain Δ*lenB2*-Δ*pheA*. -: negative control, H_2_O was used as the template. D: the gDNA of Δ*lenB2*-Δ*pheA* was used as the template by external primers *pheA*-UF/ *pheA*-DR (a DNA fragment of 2759 bp was amplified) and internal primers *dasR*-VFI/*dasR*-VRI (no DNA fragment was amplified). W: positive control, the gDNA of Δ*lenB2* was used as the template by external primers *pheA*-UF/ *pheA*-DR (a DNA fragment of 3692 bp was amplified) and internal primers *pheA*-VFI/ *pheA*-VRI (a DNA fragment of 431 bp was amplified). M: DNA marker. U, the upstream region of *pheA*; D, the downstream region of *pheA*.


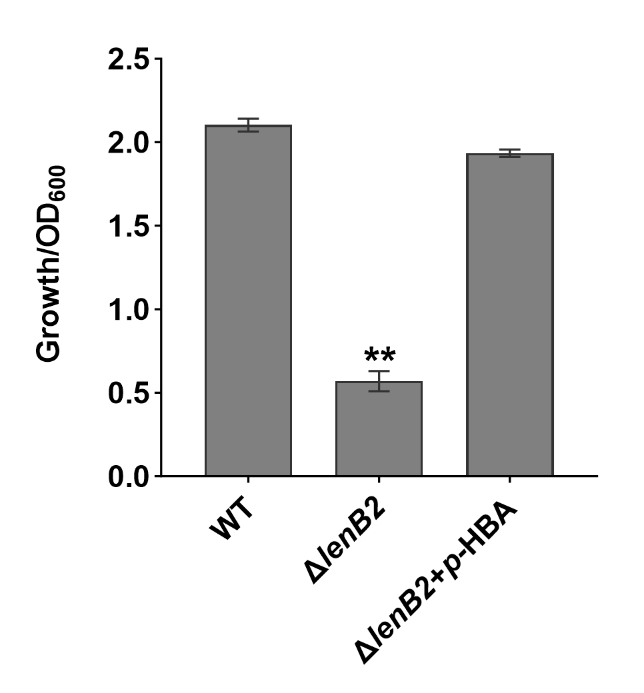


**Figure S30** The OD_600_ value of WT, Δ*lenB2* and Δ*lenB2* with *p*-hydroxybenzaldehyde (*lenB2*+*p*-HBA) grown in M813m-Fe medium for 72 h. WT was used as the control strain. Data are presented as averages of three independent experiments, each conducted in triplicate. Statistical analyses were conducted using one-way analysis of variance (ANOVA) followed by Tukey’s post-hoc test. ***p* < 0.01


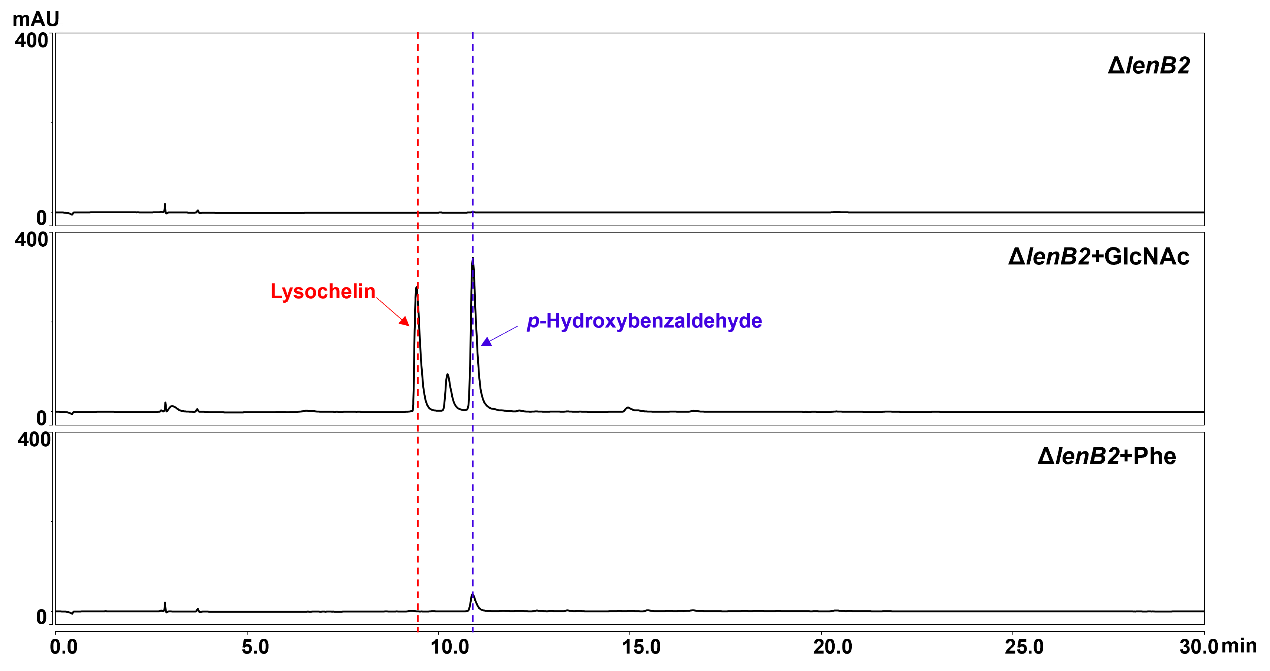


**Figure S31** HPLC analysis of lysochelin and *p*-hydroxybenzaldehyde production in Δ*lenB2*, Δ*lenB2* with GlcNAc (Δ*lenB2*+GlcNAc) and Δ*lenB2* with L-phenylalanine (Δ*lenB2*+Phe) grown in M813m-Fe medium for 72 h.


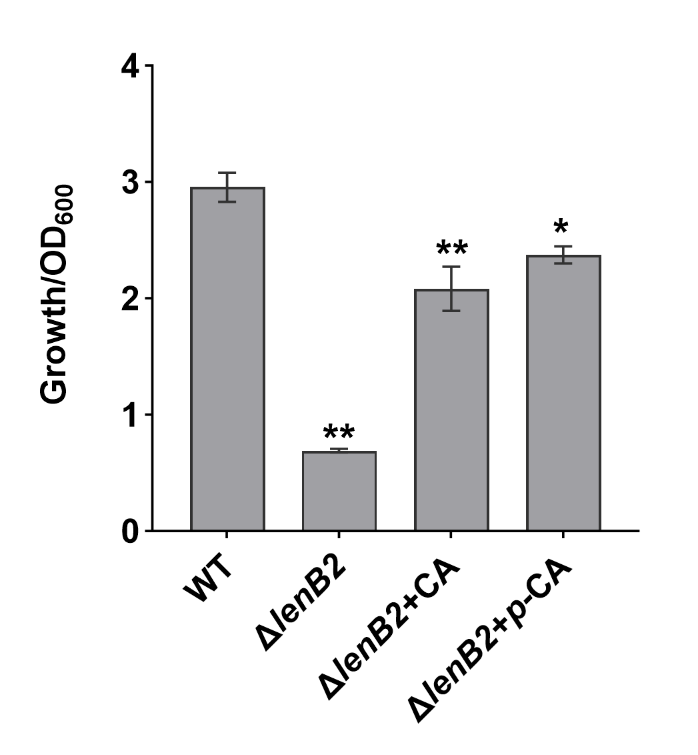


**Figure S32** The OD_600_ value of WT, Δ*lenB2*, Δ*lenB2* with cinnamic acid (Δ*lenB2*+CA) and Δ*lenB2* with *p*-hydroxycinnamic acid (Δ*lenB2*+*p*-CA) grown in M813m-Fe medium for 72 h. WT was used as the control strain. Data are presented as averages of three independent experiments, each conducted in triplicate. Statistical analyses were conducted using one-way analysis of variance (ANOVA) followed by Tukey’s post-hoc test. **p* < 0.05; ***p* < 0.01


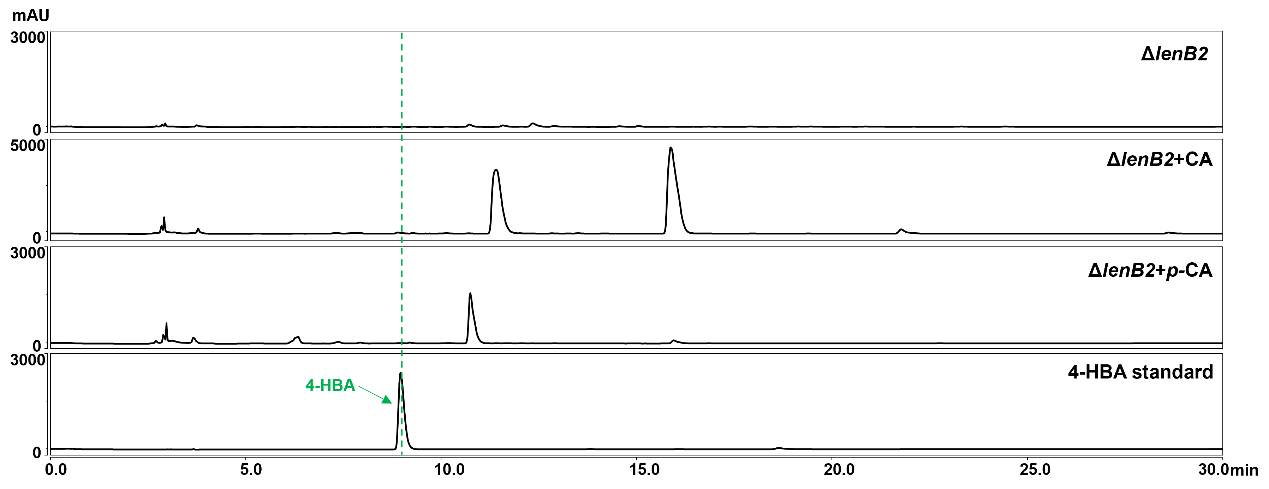


**Figure** **S33** HPLC analysis of 4-HBA production in Δ*lenB2*, Δ*lenB2* with cinnamic acid (Δ*lenB2*+CA) and Δ*lenB2* with *p*-hydroxycinnamic acid (Δ*lenB2*+*p*-CA) grown in M813m medium for 72 h, pure 4-HBA was used as standard.
